# Eliminating the VGlut2-Dependent Glutamatergic Transmission of Parvalbumin-Expressing Neurons Leads to Deficits in Locomotion and Vocalization, Decreased Pain Sensitivity, and Increased Dominance

**DOI:** 10.3389/fnbeh.2018.00146

**Published:** 2018-07-18

**Authors:** Diana M. Roccaro-Waldmeyer, Franck Girard, Daniele Milani, Elisabetta Vannoni, Laurent Prétôt, David P. Wolfer, Marco R. Celio

**Affiliations:** ^1^Anatomy and Programme in Neuroscience, Department of Medicine, University of Fribourg, Fribourg, Switzerland; ^2^Division of Functional Neuroanatomy, Institute of Anatomy, Department of Medicine, University of Zurich, Zurich, Switzerland; ^3^Institute of Human Movement Sciences and Sport, Department of Health Sciences and Technology, ETH Zurich, Zurich, Switzerland; ^4^Neuroscience Center Zurich, University of Zurich and ETH Zurich, Zurich, Switzerland

**Keywords:** parvalbumin, VGlut2, Cre/Lox-system, pain, ultrasonic vocalization, dominance, defense, parvafox nucleus

## Abstract

The calcium-binding protein parvalbumin (PV) is a recognized marker of short-axon GABA-ergic neurons in the cortex and the hippocampus. However in addition, PV is expressed by excitatory, glutamatergic neurons in various areas of the brain and spinal cord. Depending on the location of these neurons, loading of their synaptic vesicles with glutamate is mediated by either of three vesicular glutamate transporters (VGlut): VGlut1, VGlut2, or VGlut3. Driven by our interest in one of these glutamatergic/PV-expressing cell clusters—the lateral hypothalamic parvafox nucleus—we investigated the functions of this population of neurons by the selective deletion of VGlut2 expression in PV-expressing cells according to the Cre/Lox-approach. PV-Cre;VGlut2-Lox mutant mice are phenotypically characterized by deficits in locomotion and vocalization, by a decreased thermal nociception, and by an increased social dominance. We conducted a search of the Allen Brain Atlas for regions that might co-express the genes encoding PV and VGlut2, and that might thus contribute to the manifestation of the observed phenotypes. Our survey revealed several structures that could contribute to the deficits in locomotion and vocalization, such as the red, the subthalamic and the deep cerebellar nuclei. It also disclosed that a shift in the balance of afferental glutamatergic neurotransmission to the periaqueductal gray matter might be accountable for the decrease in sensitivity to pain and for the increase in social dominance. As a whole, this study broadens the state of knowledge about PV-expressing excitatory neurons.

## Introduction

Although the calcium-binding protein parvalbumin (PV) is predominantly expressed in inhibitory, GABA-ergic neurons (Celio and Heizmann, 1981; Celio, 1986, 1990; Kawaguchi et al., 1987; Röhrenbeck et al., 1987; Stichel et al., 1988; DeFelipe et al., 1989; Demeulemeester et al., 1989, 1991; Hendry et al., 1989; Blümcke et al., 1990; Lewis and Lund, 1990; Van Brederode et al., 1990; Williams et al., 1992), its presence has also been demonstrated in specific populations of excitatory, glutamatergic neurons. Among these populations are somatosensory afferents for epicritical sensibility (Celio, 1990), corticostriatal nerve cells (Jinno and Kosaka, 2004), thalamic relay-neurons (Jones and Hendry, 1989; Jones, 2001; Tanahira et al., 2009), motor and visual areas of the neocortex (Tanahira et al., 2009), and the core region of the parvafox nucleus in the lateral hypothalamus (Girard et al., 2011; Mészár et al., 2012).

Three different types of vesicular transporters exist to load synaptic vesicles with glutamate: VGlut1, VGlut2, and VGlut3. Whereas VGlut1 is the prevalent vesicular glutamate transporter in telencephalic regions, such as the cerebral cortex and the hippocampus, VGlut2 is abundant in subcortical structures, such as the thalamus, the hypothalamus, and the brainstem (Kaneko and Fujiyama, 2002; Liguz-Lecznar and Skangiel-Kramska, 2007; El Mestikawy et al., 2011).

In addition to one of these three VGlut transporters, some populations of nerve cells co-express markers of GABA-neurotransmission [e.g., glutamate-decarboxylase (GAD) and vesicular GABA-transporter (VGaT); (Docherty et al., 1987; Boulland et al., 2004; Fremeau et al., 2004; Conti et al., 2005; Seal and Edwards, 2006; El Mestikawy et al., 2011; Vaaga et al., 2014)].

Most of the PV-expressing neurons in the somatosensory cortex that project to the striatum are glutamatergic and have been demonstrated to express VGlut1 (Jinno and Kosaka, 2004). On the other hand, the PV-expressing neurons in the core region of the parvafox nucleus have been demonstrated to express VGlut2 (Girard et al., 2011; Mészár et al., 2012). In addition, PV-expressing neurons in various thalamic relay neurons (Jones and Hendry, 1989; Jones, 2001; Lewis et al., 2001; Rotaru et al., 2005; Tanahira et al., 2009; Timbie and Barbas, 2015) and in the auditory calyx of Held (Felmy and Schneggenburger, 2004) presumably express VGlut2 as well. VGlut3 is expressed in interneurons of the cortex and hippocampus (Fremeau et al., 2004), typically known to express GABA and parvalbumin. Studies directly demonstrating a coexistence of PV and VGlut2 in neurons are largely lacking. We decided to use a different approach toward bridging this gap in knowledge.

In the present study, we investigated the phenotypic consequences of eliminating the expression of VGlut2 specifically in PV-expressing neurons using the Cre/Lox-approach. A neuron's ability for glutamatergic neurotransmission depends on the presence of the respective vesicular glutamate transporter in its synaptic membrane. Accordingly, in our Cre/Lox-mouse line, any glutamatergic neuron that expresses PV and that depends solely on VGlut2 to transport glutamate into synaptic vesicles will be deficient with regard to glutamatergic neurotransmission.

A whole battery of behavioral tests were applied to the PV-Cre;VGlut2-Lox mutant mice. Since our study was originally driven by our interest in exploring the functions of the hypothalamic parvafox nucleus, these behavioral tests were mainly selected based on the connections of the parvafox nucleus with the periaqueductal gray matter (PAG). These connections preferentially indicate a possible involvement of the parvafox nucleus in analgesia, vocalization, and defense circuits (Celio et al., 2013; Bilella et al., 2016; Szabolcsi et al., 2017). In addition, a study that evaluated the effects of interfering with the fast synaptic inhibition onto PV-expressing neurons (Leppä et al., 2011) has also contributed to our selection of behavioral tests.

The aim of the present study was to correlate the behavioral phenotype of the PV-Cre;VGlut2-Lox mutant mice with the regions of the brain that may contain neurons co-expressing PV and VGlut2.

## Materials and methods

### Mice

*B6;129P2-Pvalb*^*tm*1(*cre*)*Arbr*^*/J* mice (hereafter called “PV-Cre”), expressing the Cre-recombinase gene under the control of the *Pvalb* promoter were kindly provided by Silvia Arber (FMI, Basel, Switzerland) and also purchased from the Jackson Laboratory (Bar Harbor, ME, USA). *Slc17a6*^*tm*1*Lowl*^*/J* mice (hereafter called “VGlut2-Lox”), carrying a floxed *Slc17a6* gene were kindly provided by Bradford Lowell (Harvard University, Boston, USA) (Tong et al., 2007). We crossed these two mouse strains to generate *Pvalb*^*Cre*/+^*; Slc17a6*^*Lox*/*Lox*^ mice. Crossing these latter, we generated *Pvalb*^*Cre*/*Cre*^*; Slc17a6*^*Lox*/*Lox*^ mice (hereafter referred to as “*KO*”) lacking VGlut2 protein expression in PV-expressing cells, *Pvalb*^*Cre*/+^*; Slc17a6*^*Lox*/*Lox*^ mice (hereafter referred to as “*HET*”), in which we expect a lower Cre expression and thus lower recombinase activity, and *Pvalb*^+/+^*; Slc17a6*^*Lox*/*Lox*^ mice (hereafter referred to as controls [“*CTR*”]). Mice were genotyped using classical PCR techniques according to protocols available from the supplier. Mice had free access to water and rodent chow and rooms were maintained under a 12 h light/dark cycle where not otherwise mentioned. This study was carried out in accordance with the institutional guidelines of the University of Fribourg. The protocol was approved by the Veterinary Commission for Animal Research of the Canton of Fribourg (permission nr. 2010-27-Fr) or of the Canton of Zurich (ZH 204/2008 and 29/2012).

### Western blot of brain extracts

We isolated brains of mice of all three genotypes (*CTR*/*HET*/*KO*) and homogenized them after removal of the telencephalon and cerebellum. We extracted soluble proteins for Western blotting as described elsewhere (Maetzler et al., 2009). We used SDS-PAGE (10%) to separate proteins (40 μg). After electrophoresis, we transferred the proteins onto nitrocellulose membranes (MS solution, Chemie Brunschwig, Basel, Switzerland) and blocked the membranes for 30 min at room temperature in 5% non-fat milk in TBS before incubating them overnight at 4°C with the primary antibody rabbit anti VGlut2 (Synaptic Systems, Göttingen, Germany), diluted 1:5,000 or 1:10,000 in 2% non-fat milk in TBS. After three rinses in TBS, we incubated the membranes for 2 h with HRP conjugated goat anti-rabbit IgG (Sigma-Aldrich, Buchs, Switzerland) diluted 1:7,000 in TBS. We rinsed the membranes three times, developed them using ECL technology (Merck Millipore, Schaffhausen, Switzerland) and quantified the bands with Alpha VIEW SA software (California, USA). For normalization, we used the integral of the protein signals per sample of the Ponceau Red-stained membranes.

### Detection of VGlut2 by immunofluorescence

The immunofluorescence technique was performed as previously described (Gerig and Celio, 2007; Mészár et al., 2012). In short, CTR, HET, and KO mice (*n* = 3/genotype) were perfused with 0.9% NaCl, followed by 4% paraformaldehyde, diluted in 0.1M phosphate buffered saline, pH 7.3. The brains were dissected and immersed in 30% sucrose for one night, and then frozen on dry ice. Eighty micrometers of thin cryomobile (Reichert Jung) sections were prepared and incubated in parallel in 24-well plates with the primary antiserum against VGlut2 (Synaptic Systems, Göttingen, Germany), diluted 1:10,000 to 1:20,000. The efficacy and specificity of this antiserum against VGlut2 was established by immunoblotting experiments (see section Western Blot of Brain Extracts). Incubation with the biotinylated secondary antibody was followed by exposure to streptavidine-Cy3 (Alexa 550). The sections were mounted on glass slides for histological inspection in either a Leica 6,000 epifluorescence microscope (equipped with a Hamamatsu C4742-95 camera) or a digital slide-scanner (Nanozoomer, Hamamatsu).

### Potential co-expression of the *Pvalb, Slc17A6, GAD, and Slc32A1* genes

*In-situ* hybridization images from corresponding levels available in the Allen Brain Atlas (ABA, http://mouse.brain-map.org) (Lein et al., 2007) for the expression of *Pvalb*-mRNA (ABA-experiment RP_071204_01_E06) and *Slc17a6* mRNA (the gene encoding VGlut2 protein; ABA-experiment RP_050921_01_E03) were compared with the aim to detect neurons potentially co-expressing both mRNAs. Each candidate area was listed, the number of a section containing the respective area in the two experiments was indicated, and the overall intensity of labeling in this area was rated [+ until +++] for both genes. The evaluation took into consideration also the shape, size, and distribution of the neurons. The same approach was chosen to detect coexistence of *Pvalb/Slc17a6* with the expression of *GAD* and *Slc32a1* (coding VGaT), two markers for GABAergic neurotransmission.

### Behavioral experiments

Behavioral experiments were conducted either at the University of Fribourg or after transportation of the animals and acclimatization for at least 1 week, at the Institute of Anatomy of the University of Zurich. The experiments described in this study involved a total of 124 adult mice of both sexes: 55 *CTR* (21*M*, 34*F*), 33 *HET* (19*M*, 14*F*), and 36 *KO* (18*M*, 18*F*). However, every single experiment involved only a subgroup of mice, usually of both sexes. The general health of the mice was closely monitored and mice showing signs of weakness or disease were excluded from the experiments and sacrificed. Mice were weighed regularly throughout the experiments and the mean of all measurements (1–27 measurements per mouse) was calculated for every mouse.

#### Nest assessment

A group of individually housed mice (12 *CTR* [6*M*, 6*F*], 13 *HET* [8*M*, 5*F*], and 6 *KO* [3*M*, 3*F*]) was provided in the evening with one piece of Kleenex® but no other environmental enrichment items. The next morning, photographs were taken of every cage from three different directions of view to allow for a blinded observer to rate the quality of individual nests built overnight on a scale from 1 to 5 as described elsewhere (Deacon, 2006).

#### Open field

A cohort of group-housed mice (10 *CTR* [5*M*, 5*F*], 9 *HET* [6*M*, 3*F*], and 10 *KO* [5*M*, 5*F*]) was subjected to an open field test in an empty polypropylene cage (550 mm long × 370 mm wide × 200 mm high). Mice were placed into the cage center and their behavior was recorded during 5 min from above by a video camera. The occurrence of rearing and grooming was recorded manually during the test, the number of fecal boli deposited by each mouse was counted at the end of the 5 min and locomotor activity was estimated from the movies as the number of cage line crossings (2 lines in both dimensions, separating the cage into a total of 9 rectangles). Line crossings were analyzed off-line using Observer XT 11 (Noldus Information Technology, Wageningen, Netherlands).

#### Hot plate

To assess pain sensitivity, two different groups of mice were used. Hot plate 1 was conducted at the University of Zurich on the same group of 31 mice already used for nest assessment. Hot plate 2 was conducted at the University of Fribourg on a group of mice almost identical to the one tested in open field (7 *CTR* [4*M*, 3*F*], 9 *HET* [6*M*, 3*F*], and 8 *KO* [4*M*, 4*F*]). Mice were placed in a glass chamber mounted on a hot plate maintained at 52 ± 0.1°C (IITC Life Science, Woodland Hills, CA, USA). Latency to shaking and licking of hindpaws as well as to jumping was recorded. Hindpaw licking and jumping served as experimental endpoints, but if neither of the two occurred within 40 s (cut-off time), the mouse was removed from the plate to prevent tissue damage.

#### Vocalization experiments

##### Dyadic interactions between pairs of genotype-matched female mice

Out of the 20 female mice already used for ActiviScope and individually housed over several weeks, four per genotype with similar weights were chosen for this experiment. Pairs of genotype-matched mice (weight range: 15–25 g, age range 8–9 months) were allowed to interact together during 5 min, while behavior was recorded by a video camera installed from above. Every mouse served as the resident in one interaction and as the intruder in a second one, thus there was a total of four interactions per genotype. Ultrasonic vocalizations were acquired by an ultrasonic condenser microphone (CM16/CMPA, Avisoft Bioacoustics, Berlin, Germany), located at constant distance from the cage. The acoustic signals were amplified and then digitized at 300 kHz with 16-bit resolution (UltraSoundGate 116H, Avisoft Bioacoustics) and displayed on a computer in real-time with Recorder_USGH software (Avisoft Bioacoustics). Off-line analysis of behavior was performed with Observer XT 11 by recording social investigation of the intruder by the resident to obtain total durations of anogenital, body and nose-to-nose sniffing for every interaction. The ultrasonic vocalizations (USV) emitted by the genotype-matched pairs were analyzed off-line using SASLab Pro 5.1 (Avisoft Bioacoustics) by manually selecting individual elements. Spectrograms were produced with the following parameters: FFT length = 512, frame size = 75%, window: hamming, overlap = 50%, and a cut-off frequency of 33 kHz was set to eliminate insignificant low-frequency noise. Call activity (the number of USV per minute of interaction) was determined as well as mean USV duration, mean peak frequency (the frequency at maximal amplitude within each USV) and mean peak amplitude (maximal amplitude per USV). In addition, USV were classified according to rules slightly modified from previous work of two other groups (Scattoni et al., 2008; Grimsley et al., 2011).

##### Dyadic interactions between pairs of genotype-matched male mice

A total of 15 male mice (5 *CTR*, 5 *HET*, 5 *KO*; weight range: 21–30 g and age range 8–9 months for 4 *CTR*, 4 *HET*, 3 *KO*; 18–22 g and 3–3.5 months for 1 *CTR*, 1 *HET*, 2 *KO*) were tested in genotype-matched dyadic interactions. As these *males* were group-housed, all but one mouse were removed from the cage of the resident mouse, and a genotype-matched intruder mouse from a different cage and litter was inserted. Every individual mouse was used in 3–4 interactions (1 *HET* in only two) and as both resident and intruder. As described above for *females*, behavior and USV were recorded during 5 min. In case of a fight lasting at least 10 s, one *male* was removed for another 10 s and then reintroduced into the cage. Ultrasonic vocalizations produced by each pair were analyzed as described above, and Observer XT 11 served to register fight occurrence and obtain a total duration of fights for every interaction.

##### Dyadic interactions between pairs of genotype-matched mice of opposite sexes

Another group of 23 mice (*7CTR* [4*M*, 3*F*], 9 *HET* [5*M*, 4*F*], 7 *KO* [5*M*, 2*F*]; weight range: 16–26 g and age range 3–4 months) was used for dyadic interactions between a *male* (resident) and a *female* (intruder) of the same genotype. *Males* as well as *females* were group-housed, thus all cage mates were again removed from the cage of the resident and a genotype-matched intruder female from a different litter was inserted for 5 min. Every male was used once as the resident, whereas individual females were used as intruder in one to three interactions. This experiment thus involved a total of 14 interactions (4 CTR, 5 HET, 5 KO) and it was repeated 5 times with intervals of at least 2 days in between in an attempt to equalize estrous cycle phases of all females throughout the experiment. Mouse pairs were identical across the 5 experimental days, but the order of genotypes within every block of three interactions was varied systematically (*KO*-*HET*-*CTR* on days 1 and 2, *CTR*-*HET*-*KO* on days 3 and 4, *HET*-*KO*-*CTR* on day 5) to rule out any effects of testing order. Again, behavior and USV were recorded during 5 min and USV analyzed as described above. No action was taken when *males* mounted *females* or obviously tried to do so, but such behavior was recorded using Observer XT 11 and a total duration of mounting attempts was obtained for every interaction. In addition to call activity, cumulative USV duration was calculated as the summed duration of all individual vocalizations produced by each pair within the 5 min.

#### Tube test: male dominance hierarchy

A group of *male* mice, already used for hot plate 2 and vocalization experiments (4-5 *CTR*, 4 *HET*, and 3-5 *KO*), was tested for social hierarchy in the tube test as described by others (Wang et al., 2011). As mice were rather too small for the tube, a ruler was used to separate the internal diameter into two compartments, which prevented the two cagemates from crossing each other. The same pairs were tested on a total of 7 experiment days. Experiment days 1–3 were before hot plate 2, days 4–6 were after hot plate 2, and the last test was conducted on experiment day 7 after the dyadic interactions between male mice to check whether fights may have caused any changes in social dominance hierarchy. Within each cage of 3–5 *males*, mice were ranked by their winning times that could vary between 0 and 3 (2–3 cages) or 0 and 5 (1 cage). To make ranks comparable, the 5 mice of the larger cage could obtain ranks 1.5 and 2.5 in addition to 1, 2, and 3. Tube test data were analyzed in two different ways: a (genotype × time) ANOVA was run to investigate genotype and time effects on the obtained ranks and a chi-square test was used to detect a possible dependence of the percentage of interactions won on genotype.

### Statistics

For the analysis of behavioral experiments that involved male as well as female mice, we ran two-way analyses of variance (ANOVAs) with genotype (*CTR/HET/KO* or *CTR/MUT*) and sex (*M/F*) as between-subjects factors. For the analysis of behavioral experiments that involved only males, only females, or only pairs of opposite sexes, we ran one-way ANOVAs with genotype (*CTR/HET/KO*) as between-subjects factor. For the analysis of behavioral experiments that involved several experimental phases (three-chamber test, IntelliCage, Morris water maze), we used experimental phase as additional between-subjects factor. A Tukey test was chosen for *post-hoc* analysis of all ANOVAs to compare genotypes. Statistical tests other than ANOVAs are explicitly stated in the Results section (three-chamber test: *T*-tests; tube test: chi square test; vocalization experiments: correlation analyses). IBM SPSS Statistics 21.0 was used for all statistical analyses and to create graphs. The whisker ends in boxplots represent the most extreme values, still within 1.5 inter-quartile ranges of the quartiles displayed in the boxes. Circles in boxplots represent outliers (distance from the boxes: 1.5–3 interquartile ranges), whereas asterisks represent extreme values (distance from the boxes: >3 interquartile ranges). Normality was assumed for all comparisons of group means. Statistical significance was set at *p* < 0.05.

## Results

### Western blot (VGlut2 protein quantification)

**Figure 1:**
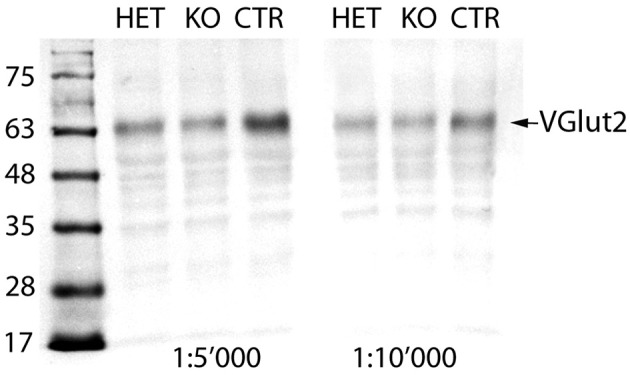
Immunoblot of brain extracts: In the brain extracts of all three genotypes, the VGlut2 antiserum recognizes a band at MW of ~64 kDa, corresponding to the VGlut2 protein (arrow). The intensity of the band is stronger in the *CTR* than in both *HET* and *KO* mice. Quantification of the band densities with Alpha VIEW software yielded mean quantities of the VGlut2 protein in the *KO* of 82.7% and in the *HET* of 86.2% of the *CTR* values (set to 100%).

Averaging the quantities of the VGlut2 protein calculated for the two different conditions (concentrations of the primary antibody), the mean quantity of the VGlut2 protein in the *KO* mouse reached 82.7% and in the *HET* mouse 86.2% of the *CTR* mouse values (set to 100%; Figure 1). It seems reasonable that 83% of VGlut2 protein remain expressed in the brain of a *KO* mouse, as most neurons that express VGlut2 are not expected to express PV, and VGlut2 expression has thus remained intact in these neurons.

### Detection of VGlut2 by immunofluorescence

**Figure 2:**
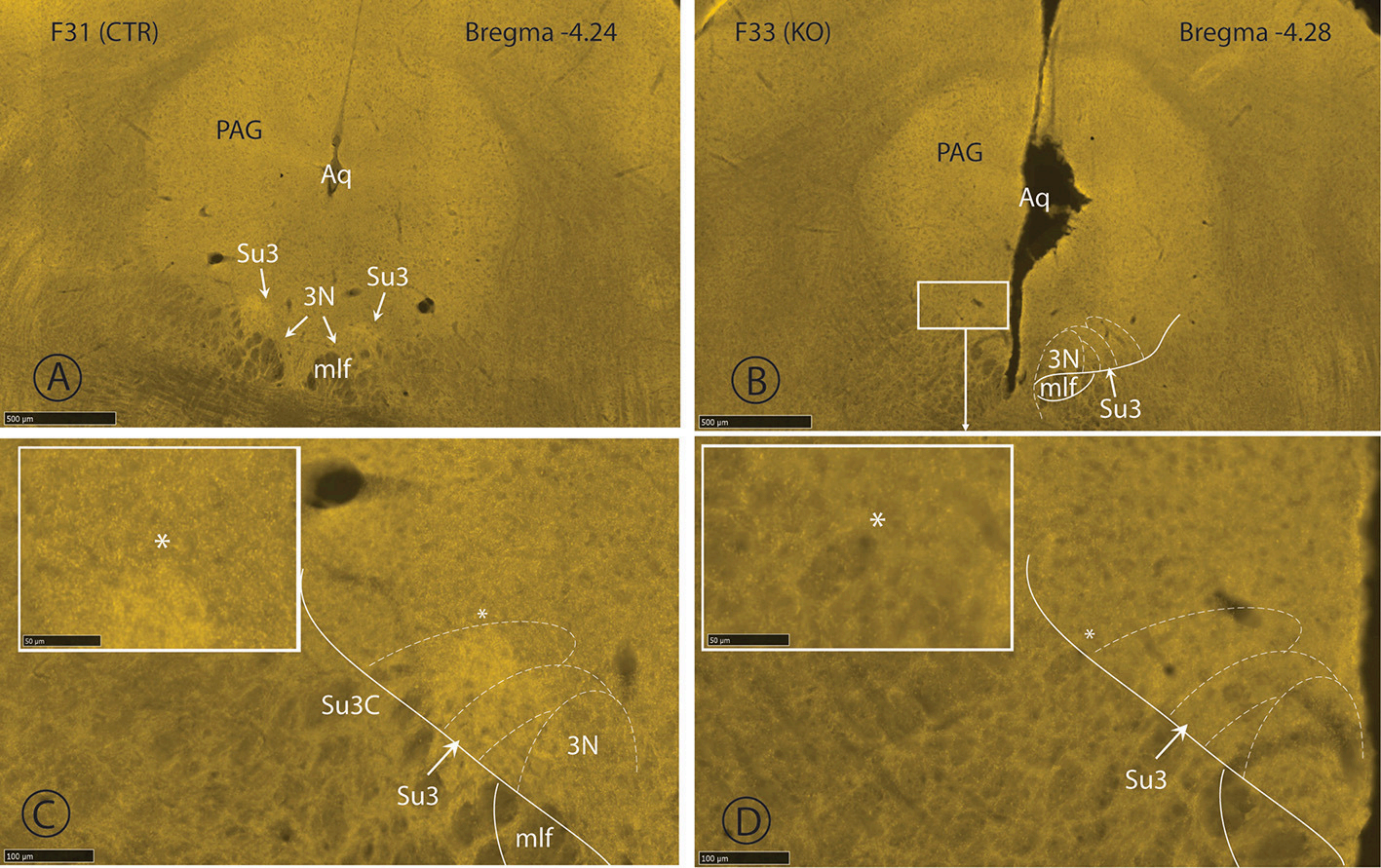
Immunfluorescence staining with the VGlut-2 antiserum. The two upper nanozoomer images **(A,B)** show a low magnification of the periaqueductal gray, with the Su3-region demarcated on the right in *CTR* (**A**; F31) and *KO* (**B**; F33) mice. The Su3 receives inputs from the PV/VGlut2 neurons of the parvafox nucleus of the lateral hypothalamus and we supposed that a difference in density of terminals could be recognized at the light microscopic level. This is indeed the case, the immunolabelling for VGlut2-terminals being more intensive in the *CTR* **(C** and terminal density in the inset) than in the *KO*-Su3 **(D** and terminal density in the inset). Aq, aqueduct; mlf, medial longitudinal fasciculus; PAG, periaqueductal gray; Su3, supraoculomotor nucleus; Su3C, supraoculomotor cap. The asterisks in the insets indicate the approximate region in **(C**,**D)**, from which the high magnification image was obtained.

The immunofluorescent staining for VGlut2 was very strong in all genotypes. In overviews, it was difficult to distinguish differences between the staining of control sections and those of HET or KO mice. The immunolabelling for VGlut2 was limited to synaptic endings, whereas perikarya remained unstained. Therefore, it was not possible to confirm the absence of VGlut2 in cell bodies of the 14 brain regions in which mRNA for *Pvalb* and *Slc17a6* may be co-expressed (see section Potential Co-expression of the *Pvalb* and the *Slc17a6* Genes and Table S1). To circumvent this disadvantage, we searched for differences in the density of terminal fields in certain regions of the brain. We centered our attention to a zone of the ventrolateral periaqueductal gray in which axons of the PV/VGlut2 neurons of the hypothalamic parvafox nucleus terminate (Celio et al., 2013). In this so called Su3 (supraoculomotor) region, the density of terminals in *CTR, HET* and *KO* brain slices was evaluated qualitatively on Nanozoomer scans (Figure 2). There was a hint of a difference in the density of VGlut2 positive endings, but a detailed morphometric analysis would be necessary to confirm this impression.

### Potential co-expression of the *Pvalb* and the *Slc17A6* genes

Screening of the Allen Brain Atlas revealed 65 brain regions in which the mRNAs of both *Pvalb* and *Slc17a6* were expressed (Table S1). In most of these regions, the pattern of distribution of the label for the two types of mRNA differed substantially. However, in 14 of the regions (highlighted in gray in Table S1), the labeling for the two genes occurred at least partially in corresponding loci or occupied the entire cross-section through the respective region. Hence, these 14 regions may—to varying extents—contain neurons that co-express the two genes. The implicated regions include (1) motor areas, such as the deep cerebellar, the red and the subthalamic nuclei, and (2) sensory ones, such as the cuneate and gracile nuclei, as well as the cochlear and vestibular nuclei, and the principal sensory nucleus of the trigeminal nerve. In addition, they include (3) the caudal portion of the pontine reticular nucleus, (4) the ventral anterior-lateral complex of the thalamus, and (5) the parvafox nucleus in the hypothalamus. Each of the phenotypic traits observed in PV-Cre;VGlut2-Lox mice (and presented hereafter) could thus arise as a consequence of eliminating the expression of VGlut2 in the neurons of any of these 14 regions.

### Potential co-expression of the *Pvalb* and the *Slc17A6* genes and markers for GABA transmission (*GAD* and *Slc32A1* genes)

To evaluate the occurrence of markers for GABA-neurotransmission in the 14 nuclei with a probable co-expression of *Pvalb* and *Slc17a6 (coding for VGlut2)*, the Allen Database was screened for the expression of *GAD* and *Slc32a1* (coding for VGaT) (Table S1). In only one brain region were these four genes expressed abundantly throughout the entire region, namely in the medial vestibular nucleus. In all other nuclei, the number of GABA and/or VGaT expressing neurons was lower or distributed differently from those expressing VGlut2 and PV.

### Phenotypic traits resulting from the elimination of *Slc17A6* expression in PV-expressing neurons

**Figure 3:**
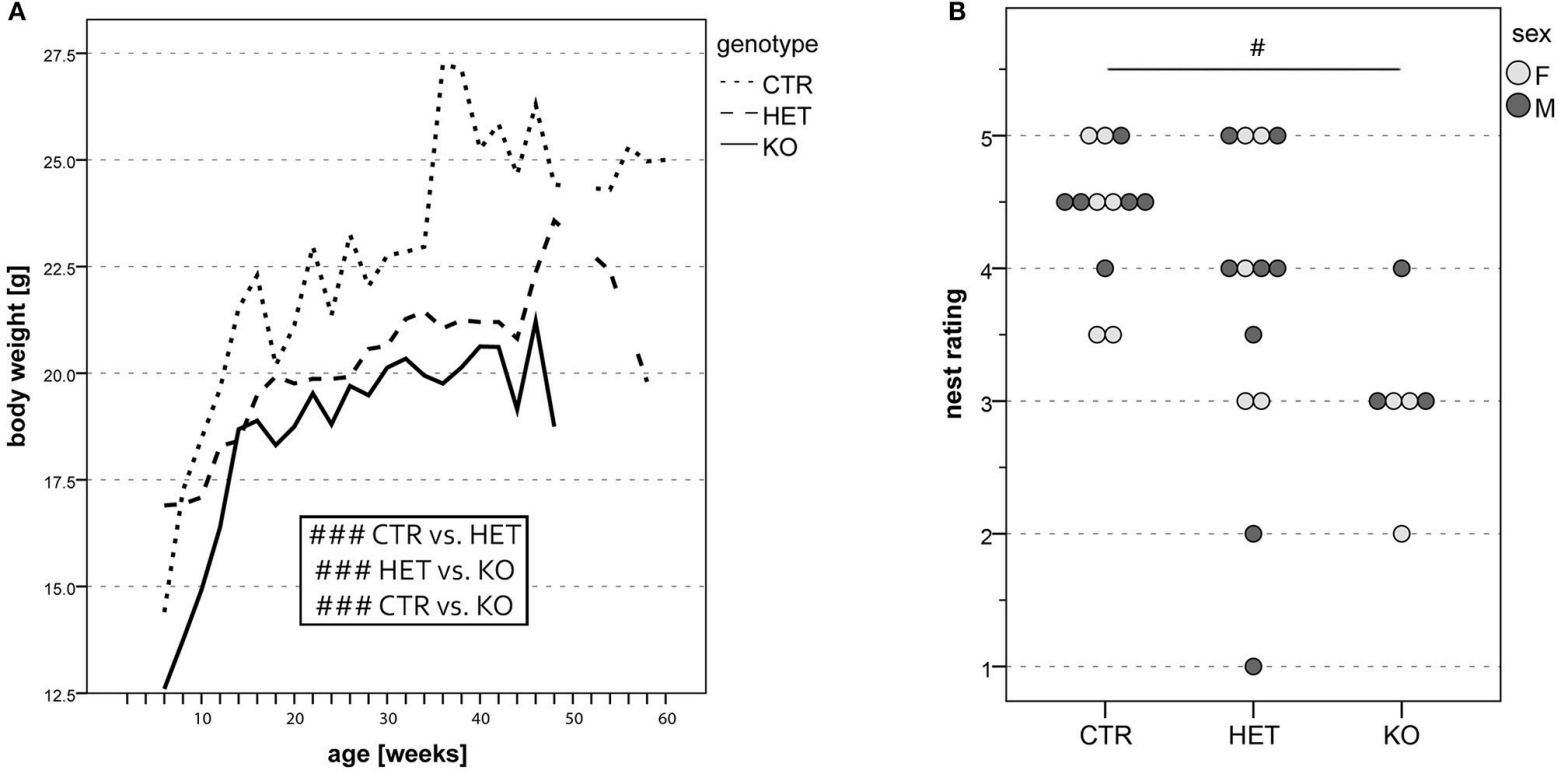
Body weight of mice at different ages. **(A)** Overall body weight was lower in both *KO* and *HET* than in *CTR* mice. *CTR* mice were heavier than *HET* mice, and the latter were heavier than *KO* mice. Furthermore, *male* mice were heavier than *female* mice. Data originate from all mice used for behavioral experiments (*n* = 55 *CTR*, 31 *HET*, 36 *KO*). Nest assessment: *KO* mice build nests of lower quality than *CTR* and *HET* mice **(B)**. Circles filled in light and dark gray represent data of single *female* and *male* mice, respectively. There was no sex difference in the quality of nests built overnight by the individually housed mice, however *CTR* mice built nests of higher quality than *KO* mice. Quality ratings were assessed by a blinded observer. (*n* = 12 *CTR*, 13 *HET*, 6 *KO*). (#*p* < 0.05, ###*p* < 0.001).

**Figure 4:**
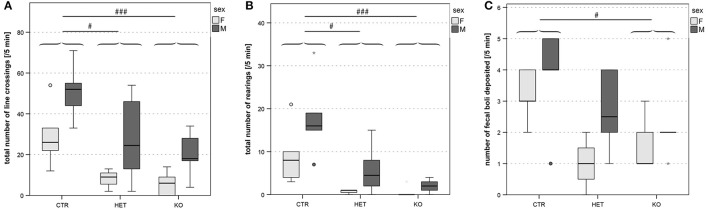
Open field: reduced locomotor- and exploratory activities and grooming. Locomotor activity, estimated from the total number of quadrant line crossings, was higher in males than in females and lower in both *KO* and *HET* than in *CTR* mice **(A)**. Vertical exploration expressed as the total number of rearing was as well lower in both *KO* and *HET* than in *CTR* mice **(B)**. The number of fecal boli deposited was lower in *KO* than in *CTR* **(C)**. Boxes filled in light and dark gray represent data of *female* and *male* mice, respectively. (*n* = 10 *CTR*, 9 *HET*, 10 *KO)*. (#*p* < 0.05, ### *p* < 0.001).

**Figure 5:**
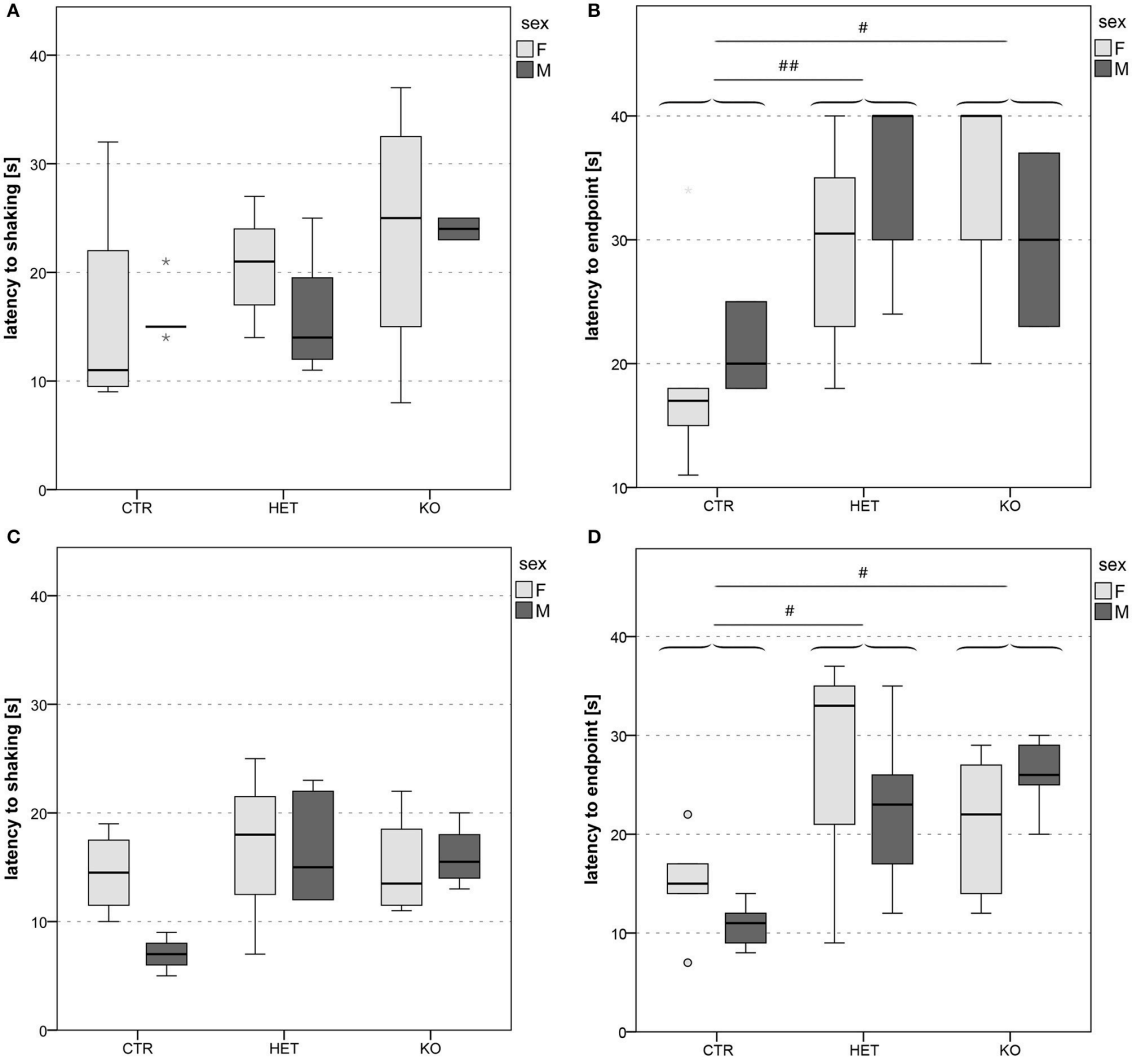
Reactivity to thermal pain: lower pain sensitivity in *KO* and *HET* than in *CTR* mice. Latency to shaking **(A,C)** and latency to endpoint **(B,D)** in hot plate 1 **(A,B)** and hot plate 2 **(C,D)**. In both cohorts of mice, latency to endpoint was longer in both *KO* and *HET* than in *CTR* mice **(B,D)**, whereas latency to hindpaw shaking did not differ between genotypes **(A,C)**. Boxes filled in light and dark gray represent data of *female* and *male* mice, respectively. (*n* = 9 *CTR*, 12 *HET*, 6 *KO* for hot plate 1, *n* = 7 *CTR*, 9 *HET*, 8 *KO* for hot plate 2). (#*p* < 0.05, ##*p* < 0.01).

**Figure 6:**
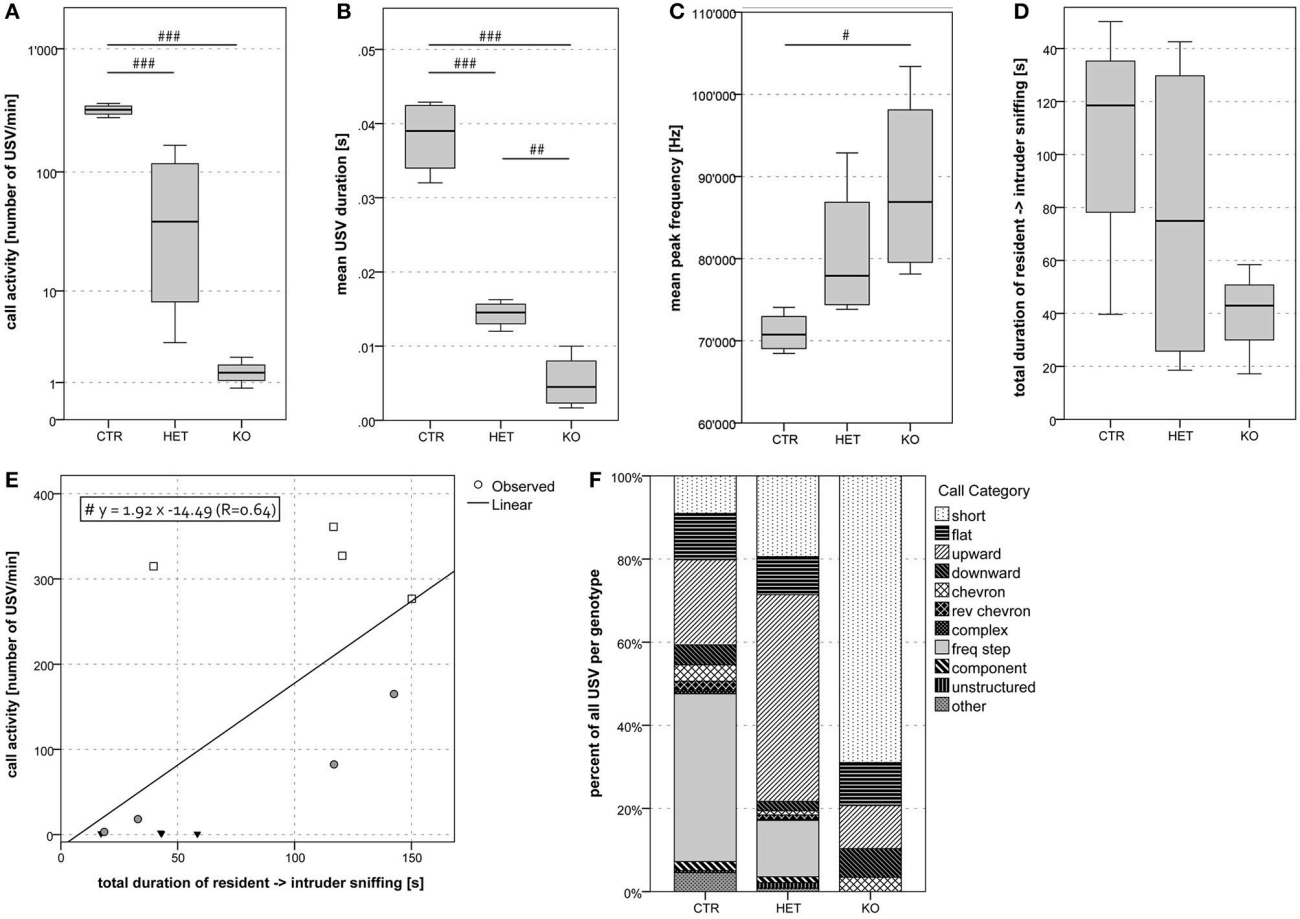
Dyadic interactions between female pairs of mice: lower frequency of vocalization and restricted repertoire in *KO*-mice. Mean call activity was lower in *KO* and *HET* than *CTR* pairs **(A)**. Mean USV duration was lowest in *KO* and lower in *HET* than *CTR* pairs **(B)**. Mean peak frequency was higher in *KO* than *CTR* pairs **(C)**. There was a trend for a higher total duration of resident → intruder sniffing (all body areas) in *KO* and *HET* than in *CTR* pairs **(D)**. In the sample as a whole, we found a positive correlation between mean call activity and the total duration of resident → intruder sniffing (all body areas) **(E)**. Black triangles, circles filled in gray and empty squares represent data of *KO, HET* and *CTR* pairs, respectively. KO pairs produced only USV belonging to the less complex types, whereas both HET and CTR pairs produced USV of the entire repertoire **(F)**. (*n* = 4 *CTR*, 4 *HET*, 4 *KO*). (#*p* < 0.05, ##*p* < 0.01, ###*p* < 0.001).

Mice in which the PV-expressing cells lacked VGlut2 (*KO* mice) had a lower body weight than their wild-type littermates (*CTR* mice, Figure 3A), and were hypoactive according to several criteria. In the open field test, hypoactivity was manifested as a reduction in locomotive and exploratory activity (Figures 4A,B). The deficits in locomotion were both qualitative and quantitative. The *KO* mice experienced difficulties in maintaining a horizontal body axis while walking and swimming (Figure S2C). In addition, they built nests of lower quality than the *CTR* mice (Figure 3B). The threshold for thermal pain appeared higher in *KO* and *HET* mice than in *CTR* mice (Figures 5B,D). *KO* mice vocalized less than *CTR* mice (Figures 6A, 8A), and female pairs of *KO* mice exclusively produced vocalizations of the less complex patterns (Figure 6F). As assessed by the tube test, *KO* and *HET* males were more dominant than *CTR* males (Figure 9). However, according to the three-chamber test, social behavior did not differ between genotypes (Figures S5A,B,C,E,F,G). Similarly, anxiety- and stress-related parameters (Figure 4C, Figures S4A–E), memory performance (Figures S2A, S3G,H), and circadian rhythms (Figure S1) did not differ consistently between the genotypes. Table 1 summarizes the statistically significant differences between the genotypes, whereas Table S2 summarizes sex differences and statistical interaction (genotype × sex).

**Table 1:** Summary of statistically significant differences detected between genotypes.

**Parameter differing between genotypes (experiment)**	***CTR* vs. *HET***	***HET* vs. *KO***	***CTR* vs. *KO***	***CTR* vs. *MUT***	***CTR:* mean ± SEM**	***HET:* mean ± SEM**	***KO:* mean ± SEM**	***MUT:* mean ± SEM**	**Figures**
Body weight [g]	>(^***^)	>(^***^)	>(^***^)		22.33 ± 0.16 (*n* = 55)	19.59 ± 0.19 (*n* = 33)	18.45 ± 0.19 (*n* = 36)		3A
Quality of nests			>(^*^)		4.417 ± 0.270 (*n* = 12)	3.781 ± 0.267 (*n* = 13)	3.000 ± 0.382 (*n* = 6)		3B
Number of line crossings (open field)	>(^*^)		>(^***^)		40.200 ± 4.499 (*n* = 10)	17.667 ± 5.030 (*n* = 9)	13.000 ± 4.499 (*n* = 10)		4A
Exploratory activity - rearing (open field)	>(^*^)		>(^***^)		13.600 ± 1.777 (*n* = 10)	3.167 ± 1.986 (*n* = 9)	1.300 ± 1.777 (*n* = 10)		4B
Grooming (open field)			>(^**^)		2.300 ± 0.216 (*n* = 10)	1.750 ± 0.241 (*n* = 9)	1.300 ± 0.216 (*n* = 10)		–
Number of fecal boli deposited (open field)			>(^*^)		3.500 ± 0.392 (*n* = 10)	1.833 ± 0.438 (*n* = 9)	2.000 ± 0.392 (*n* = 10)		4C
Latency to endpoint (hot plate 1) [s]	< (^**^)		< (^*^)		21.18 ± 2.51 (*n* = 9)	33.33 ± 2.19 (*n* = 12)	32.50 ± 3.23 (*n* = 6)		5B
Latency to endpoint (hot plate 2) [s]	< (^*^)		< (^*^)		13.33 ± 2.80 (*n* = 7)	24.50 ± 2.59 (*n* = 9)	25.25 ± 2.59 (*n* = 8)		5D
Mean call activity (F-F dyadic interactions)	>(^***^)		>(^***^)		320.000 ± 23.533 (*n* = 4)	67.200 ± 23.533 (*n* = 4)	1.450 ± 23.533 (*n* = 4)		6A
Mean USV duration (F-F dyadic interactions) [s]	>(^***^)	> (^**^)	>(^***^)		0.038 ± 0.002 (*n* = 4)	0.014 ± 0.002 (*n* = 4)	0.005 ± 0.002 (*n* = 4)		6B
Mean USV peak frequency (F-F dyadic interactions) [Hz]			< (^*^)		71,012 ± 4,252 (*n* = 4)	80,634 ± 4,252 (*n* = 4)	88,829 ± 4,252 (*n* = 4)		6C
Total duration of body sniffing (F-F dyadic interactions) [s]	>(^*^)		>(^*^)		44.502 ± 7.665 (*n* = 4)	13.043 ± 7.665 (*n* = 4)	8.013 ± 7.665 (*n* = 4)		–
Total duration of fights (M-M dyadic interactions) [s]			>(^*^)		12.118 ± 3.200 (*n* = 5)	4.261 ± 3.421 (*n* = 5)	0.000 ± 3.200 (*n* = 5)		7B
Mean call activity (F-M dyadic interactions)		>(^***^)	>(^***^)		344.20 ± 38.43 (*n* = 4)	345.52 ± 34.37 (*n* = 5)	63.86 ± 34.37 (*n* = 5)		8A
Total duration of mounting attempts (F-M dyadic interactions) [s]			>(^**^)		23.385 ± 5.052 (*n* = 4)	9.376 ± 4.519 (*n* = 5)	2.054 ± 4.519 (*n* = 5)		8B
Mean USV duration (F-M dyadic interactions) [s]	>(^*^)	>(^*^)	>(^***^)		0.044 ± 0.004 (*n* = 4)	0.029 ± 0.004 (*n* = 5)	0.017 ± 0.004 (*n* = 5)		8C
Mean USV peak frequency (F-M dyadic interactions) [Hz]			< (^*^)		76,775 ± 2,697 (*n* = 4)	84,568 ± 2,472 (*n* = 5)	86,148 ± 2,530 (*n* = 5)		8E
Mean USV peak amplitude (F-M dyadic interactions) [db]	>(^**^)		>(^***^)		−23.27 ± 1.22 (*n* = 4)	−28.46 ± 1.12 (*n* = 5)	−31.87 ± 1.15 (*n* = 5)		8F
Social dominance of males: rank obtained (tube test)	>(^***^)		>(^***^)		2.646 ± 0.120 (*n* = 5)	1.464 ± 0.128 (*n* = 4)	1.681 ± 0.130 (*n* = 5)		9A–D
Homecage activity counts (ActiviScope)	>(^***^)		>(^***^)		22.89 ± 1.44 (n = 22)	11.93 ± 1.55 (*n* = 16)	10.56 ± 2.38 (*n* = 10)		S1
Escape latency (Morris water maze, acquisition) [s]	< (^*^)		< (^**^)		73.90 ± 6.88 (*n* = 12)	99.90 ± 5.69 (*n* = 12)	107.97 ± 8.42 (*n* = 6)		S2A
Escape latency (Morris water maze, reversal) [s]	< (^***^)		< (^***^)		57.19 ± 7.40 (*n* = 12)	100.38 ± 6.13 (*n* = 12)	106.90 ± 9.07 (*n* = 6)		S2A
Swim speed (Morris water maze) [m/s]		>(^*^)	>(^***^)		0.157 ± 0.007 (*n* = 12)	0.136 ± 0.005 (*n* = 12)	0.114 ± 0.008 (*n* = 6)		S2B
Circling behavior (Morris water maze) [°]	< (^**^)		< (^***^)		586.3 ± 152.2 (*n* = 12)	1428.8 ± 126.1 (*n* = 12)	1808.5 ± 186.4 (*n* = 6)		S2C
Number of corner visits per day (IntelliCage, FAP)				>(^***^)	135.20 ± 8.73 (*n* = 11)			27.83 ± 9.66 (*n* = 9)	S3A
Number of nosepokes per corner visit (IntelliCage)				< (^***^)	5.871 ± 0.430 (*n* = 11)			11.406 ± 0.649 (*n* = 9)	S3B
Duration of corner visits (IntelliCage) [s]				< (^***^)	33.14 ± 4.68 (*n* = 11)			70.55 ± 8.04 (*n* = 9)	S3C
Number of licks (IntelliCage, EXP)				>(^***^)	1476.3 ± 87.9 (*n* = 11)			681.9 ± 115.0 (*n* = 9)	S3D
Spontaneous left/right side preferences (IntelliCage, adaptation) [%]				< (^*^)	83.30 ± 0.43 (*n* = 11)			85.27 ± 0.65 (*n* = 9)	S3E
Spontaneous corner preferences (IntelliCage, FAP) [%]				< (^***^)	34.32 ± 1.94 (*n* = 11)			47.08 ± 2.68 (*n* = 9)	S3F
Number of errors (IntelliCage, PPRA)				>(^**^)	9.725 ± 0.549 (*n* = 11)			6.375 ± 0.815 (*n* = 9)	S3G
Number of errors (IntelliCage, PPRP)				>(^***^)	13.164 ± 0.823 (*n* = 11)			7.758 ± 1.259 (*n* = 9)	S3H
Entries into open sectors (elevated zero maze)				> (^**^)	23.35 ± 2.44 (*n* = 12)			13.14 ± 2.19 (*n* = 15)	S4A
Number of stretched attend postures (elevated zero maze)				>(^**^)	1.808 ± 0.216 (*n* = 12)			1.069 ± 0.194 (*n* = 15)	S4D
Number of fecal boli deposited (elevated zero maze)				>(^**^)	4.750 ± 0.494 (*n* = 12)			2.589 ± 0.443 (*n* = 15)	S4E

*Each parameter detected to differ between genotypes is indicated in a separate line, together with the direction of its difference, statistical significance, mean ± SEM per genotype, and the respective figure. EXP, initial exploration period; FAP, free adaptation protocol; PPRA, place preference acquisition protocol; PPRP, place preference reversal protocol; >, larger/higher/longer; <, smaller/lower/shorter*;

* *p < 0.05*,

** *p < 0.01*,

*** *p < 0.001*.

#### Motricity

The two most obvious phenotypic characteristics of the PV-Cre;VGlut2-Lox mutant mice compared to their wild-type littermates were their low body weight (Figure 3A) [*F*_(2, 1, 193)_ = 140.79, *p* < 0.001; *CTR* vs. *HET p* < 0.001, *HET* vs. *KO p* < 0.001, *CTR* vs. *KO p* < 0.001] and their hypoactivity, which, in the open field (Figure 4A) and ActiviScope Tests (Figure S1) was manifested as a reduction in locomotor activity [open field, number of line crossings: *F*_(2, 23)_ = 10.26, *p* = 0.001; *CTR* vs. *HET p* = 0.02, *HET* vs. *KO p* = 0.46, *CTR* vs. *KO p* = 0.001; ActiviScope, homecage activity counts: *F*_(2, 45)_ = 17.31, *p* < 0.001; *CTR* vs. *HET p* < 0.001, *HET* vs. *KO p* = 0.73, *CTR* vs. *KO p* < 0.001]. In the open field test, *KO* and *HET* mice also reared less than *CTR* mice (Figure 4B) [rearing number: *F*_(2, 23)_ = 13.64, *p* < 0.001; *CTR* vs. *HET p* = 0.03, *HET* vs. *KO p* = 0.56, *CTR* vs. *KO p* < 0.001]; likewise, *KO* mice groomed less than *CTR* mice (not shown). In the IntelliCage test, *KO* and *HET* mice approached the corners of the cage less often than *CTR* mice (Figure S3A) [e.g., FAP: *F*_(1, 72)_ = 68.0, *p* < 0.001], but remained within its confines longer (Figure S3C) [*F*_(1, 52)_ = 16.1, *p* < 0.001]. Moreover, the *KO* mice drank an insufficient quantity of water, and thus had to be removed from the IntelliCage. On the other hand, neither in the elevated zero maze (Figure S4F) [*F*_(1, 23)_ = 1.36, *p* = 0.26], nor in the three-chamber test (Figures S5D,H) [*F*_(2, 66)_ = 1.43, *p* = 0.25; *CTR* vs. *HET p* = 0.30, *HET* vs. *KO p* = 0.56, *CTR* vs. *KO p* = 0.94], did locomotor activity, defined as the total distance traveled, differ between genotypes. In these two tests, other performance-determining factors presumably masked the genotypic differences in locomotor activity, which were obvious in the more basic tests.

Deficits in locomotion were most obvious in the *KO-*mice, but, as aforementioned, were also apparent to some extent in the *HET-*genotype. *KO-*mice had a wobbly gait, which worsened with increasing age. They tended to overbalance when walking on solid ground and were usually unable to maintain a horizontal body axis whilst swimming. This deficit probably accounted for the marked tendency of *KO-* and *HET*-mice to swim in circles (Figure S2C), rather than in straight lines, in the Morris water maze [cumulative circling: *F*_(2, 20)_ = 15.0, *p* < 0.001; *CTR* vs. *HET p* = 0.001, *HET* vs. *KO p* = 0.053, *CTR* vs. *KO p* < 0.001]. The *KO* mice that completed this test also swam significantly less rapidly than their wild-type littermates (Figure S2B) [*F*_(2, 20)_ = 8.66, *p* = 0.002; *CTR* vs. *HET p* = 0.068, *HET* vs. *KO p* = 0.031, *CTR* vs. *KO p* < 0.001].

#### Pain

One of the clearest genotypic differences was revealed by the hot plate test (Figure 5). Both the *KO-* and the *HET-*mice licked their hindpaws or jumped off the plate significantly later than *CTR-*mice (Figures 5B,D) [hot plate 1: *F*_(2, 21)_ = 7.42, *p* = 0.004; *CTR* vs. *HET p* = 0.003, *HET* vs. *KO p* = 1.00, *CTR* vs. *KO p* = 0.015; hot plate 2: *F*_(2, 18)_ = 5.98, *p* = 0.01; *CTR* vs. *HET p* = 0.036, *HET* vs. *KO p* = 0.92, *CTR* vs. *KO p* = 0.02]. Interestingly, there were no genotypic differences in the latency to hindpaw shaking (Figures 5A,C) [hot plate 1: *F*_(2, 21)_ = 1.96, *p* = 0.17; hot plate 2: *F*_(2, 18)_ = 2.83, *p* = 0.09]. This finding indicates that the three groups of mice all experienced the sensation of heat after a comparable delay, but that the threshold for the perception of thermal pain was higher in the *KO-* and the *HET*-mice than in *CTR*-mice.

#### Vocalization

**Figure 7:**
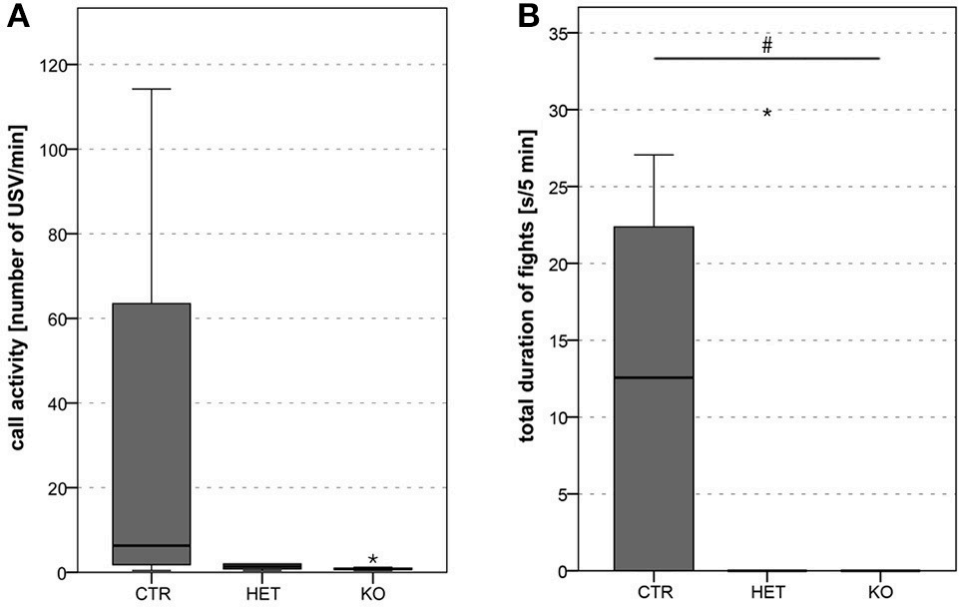
Dyadic interactions between male pairs of mice: lower fight duration in *KO* mice. Mean call activity tended to be lower in *HET* and *KO* than in *CTR* pairs **(A)**. Total duration of fights was lower in *KO* than in *CTR* pairs (HET were indifferent from both other groups due to high variability) **(B)**. (*n* = 5 *CTR*, 5 *HET*, 5 *KO*). (#*p* < 0.05).

**Figure 8:**
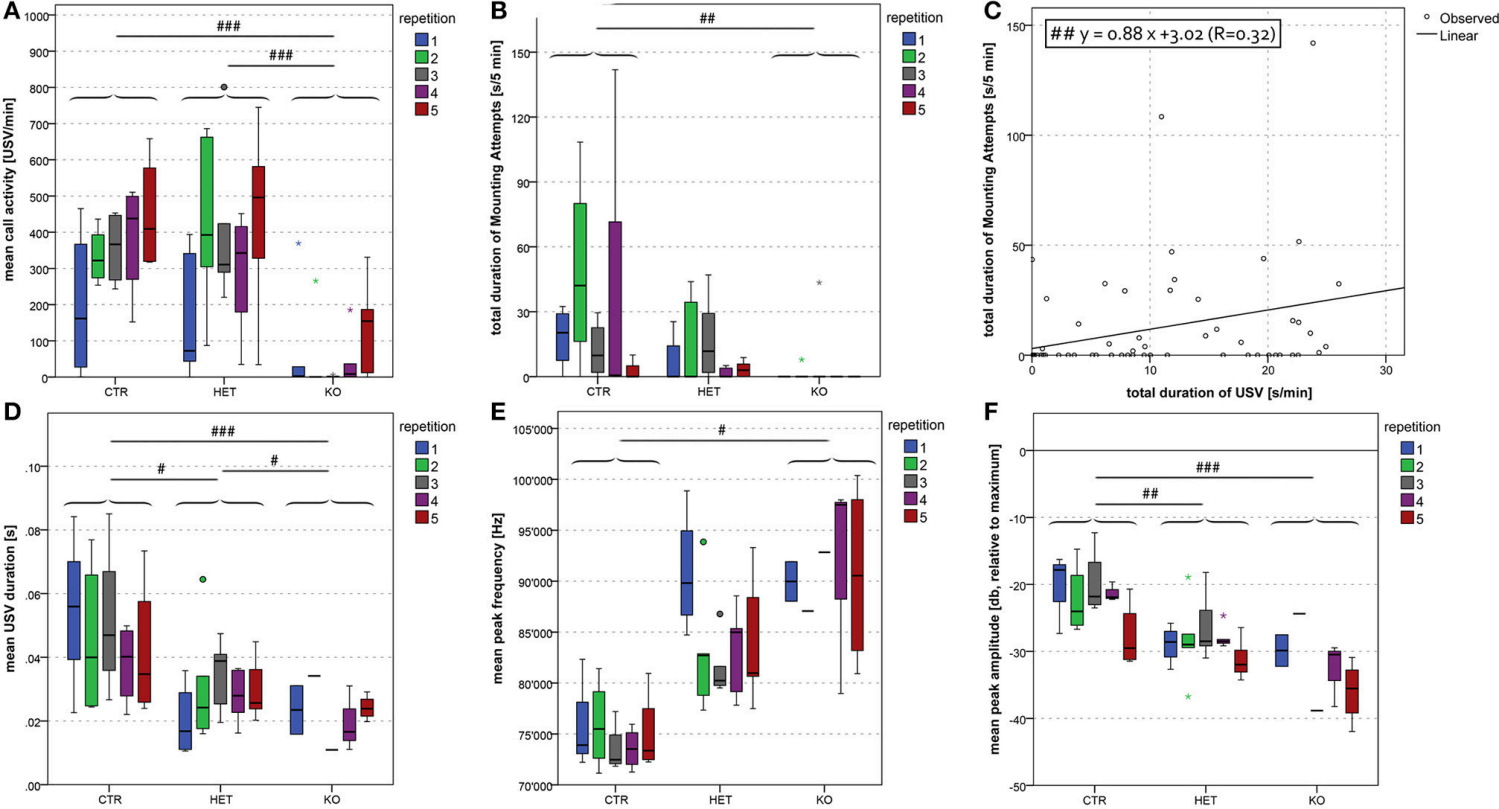
Dyadic interactions between pairs of opposite sexes: difference in duration, frequency and amplitude between *MUT* and *CTR* mice. Mean call activity was lower in *KO* than in *HET* and *CTR* pairs **(A)**. Total duration of mounting attempts was lower in *KO* than in *CTR* pairs **(B)**. In the sample as a whole, we found a positive correlation between the total duration of USV and the total duration of mounting attempts **(C)**. Mean USV duration was shorter in *KO* and *HET* than in *CTR* pairs **(D)**. Mean peak frequency was higher in *KO* than in *CTR* pairs **(E)**. Finally, mean peak amplitude was lower in *KO* and *HET* than in *CTR* pairs **(F)**. None of the parameters was significantly altered by repetition. (*n* = 7 *CTR*, 9 *HET*, 7 *KO*). (#*p* < 0.05, ##*p* < 0.01, ###*p* < 0.001).

During the dyadic interactions between two females, the frequency of the ultrasonic vocalizations that were produced (mean call activity) was lower in the *KO-* and in the *HET-* than in the *CTR-*mice (Figure 6A) [*F*_(2, 9)_ = 51.07, *p* < 0.001; *CTR* vs. *HET p* < 0.001, *HET* vs. *KO p* = 0.17, *CTR* vs. *KO p* < 0.001]. During the interactions between two males, this parameter was *tendentially* lower in the *KO-* and the *HET-* than in the *CTR-*mice (Figure 7A) [*F*_(2, 20)_ = 3.37, *p* = 0.055; *CTR* vs. *HET p* = 0.099, *HET* vs. *KO p* = 1.0, *CTR* vs. *KO p* = 0.082]. During the interactions between two females (Figure 6B) [*F*_(2, 9)_ = 80.40, *p* < 0.001; *CTR* vs. *HET p* < 0.001, *HET* vs. *KO p* = 0.019, *CTR* vs. *KO p* < 0.001] and between a female and a male (Figure 8D) [*F*_(2, 55)_ = 12.30, *p* < 0.001; *CTR* vs. *HET p* = 0.021, *HET* vs. *KO p* = 0.046, *CTR* vs. *KO p* < 0.001], but not during those between two males (not shown), the duration of the ultrasonic vocalizations was shorter in the *KO-* than in the *HET-*mice, and shorter in the latter than in *CTR*-mice. Likewise during the interactions between two females (Figure 6C) [*F*_(2, 9)_ = 4.40, *p* = 0.046; *CTR* vs. *HET p* = 0.29, *HET* vs. *KO p* = 0.40, *CTR* vs. *KO p* = 0.038] and between a female and a male (Figure 8E) [*F*_(2, 55)_ = 3.63, *p* = 0.034; *CTR* vs. *HET p* = 0.11, *HET* vs. *KO p* = 0.83, *CTR* vs. *KO p* = 0.033], but not between two males (not shown), the peak frequency of the ultrasonic vocalizations was higher in the *KO-*than in the *CTR-*mice. Genotypic differences in the peak amplitude of the ultrasonic vocalizations were observed only during the interactions between a female and a male, with that in *KO-* and *HET-*mice being lower than that in the *CTR-*mice (Figure 8F) [*F*_(2, 55)_ = 13.33, *p* < 0.001; *CTR* vs. *HET p* = 0.008, *HET* vs. *KO p* = 0.094, *CTR* vs. *KO p* < 0.001). Furthermore during the interactions between two females, mean call activity correlated with the total duration of resident → intruder sniffing (all body areas) (Figure 6E).

#### Aggression and dominance

**Figure 9:**
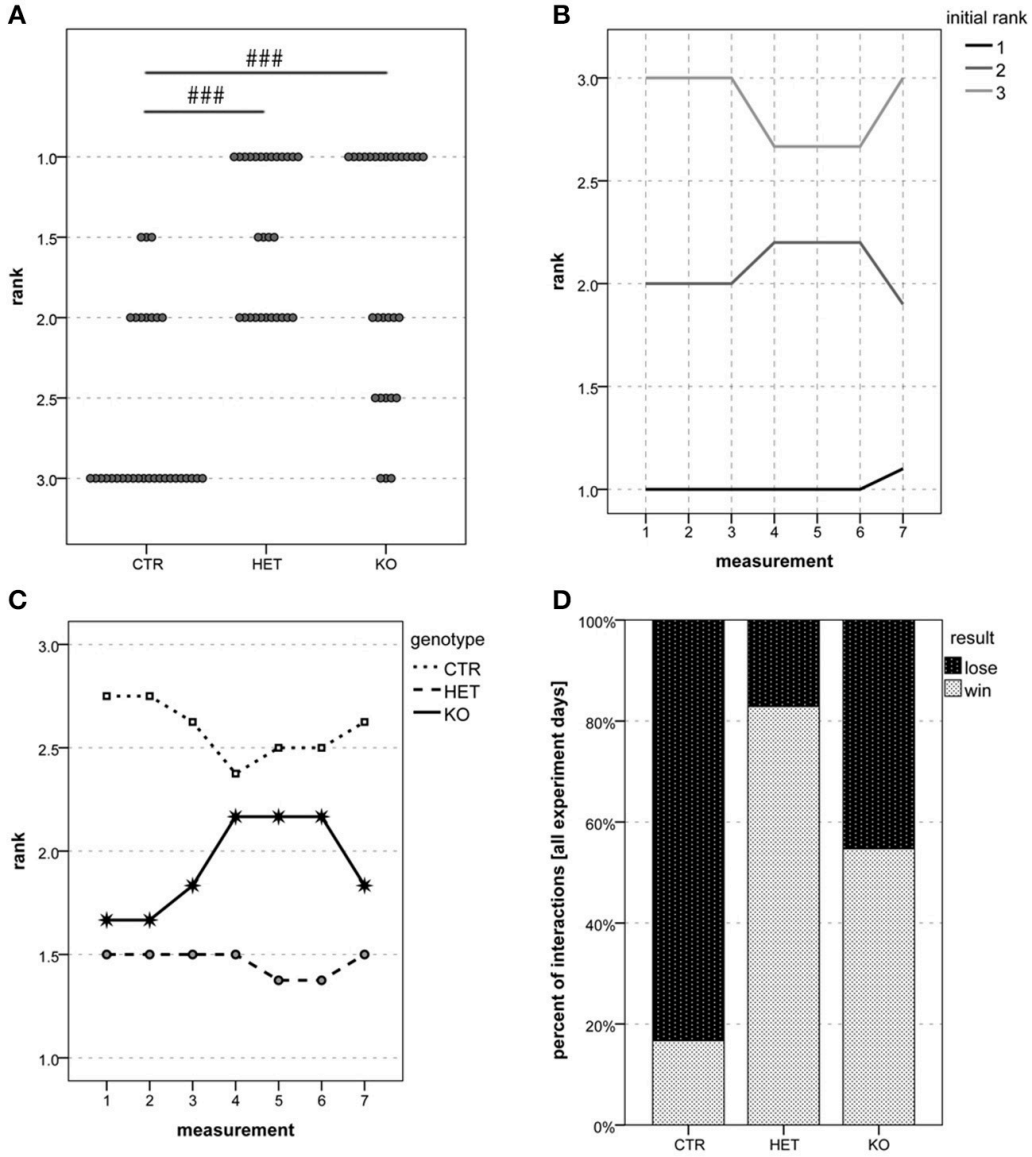
Social dominance: *HET* and *KO* mice are more dominant than *CTR* mice. In the tube test, 1 was the most common rank among both *HET* and *KO* mice, whereas 3 was the most common rank among *CTR* mice **(A)**. Ranks remained very stable over time. **(B)** shows the development of the mean rank over time for three groups of mice based on their rank reached in the first test, whereas **(C)** shows the development of the mean rank per genotype over time. Of all their interactions, *HET, KO* and *CTR* mice won 82.9, 54.7, and 16.7%, respectively **(D)**. (*n* = 5 *CTR*, 4 *HET*, 5 *KO*). (###*p* < 0.001).

During the dyadic interactions between the genotype-matched males, the readiness to fight was less marked in *KO-* than in *CTR-*mice (Figure 7B) [*F*_(2, 20)_ = 3.68, *p* = 0.044; *CTR* vs. *HET p* = 0.24, *HET* vs. *KO p* = 0.64, *CTR* vs. *KO p* = 0.037]. Whether the *KO*-males were indeed less aggressive than the *CTR-*males could not be ascertained, since the general hypoactivity of the former animals could have accounted for the findings. Inter-genotypic trials (tube test) were more often won by the *HET-* and the *KO-*mice than by *CTR-*mice (Figure 9) [*F*_(2, 68)_ = 26.1, *p* < 0.001; *CTR* vs. *HET p* < 0.001, *HET* vs. *KO p* = 0.48, *CTR* vs. *KO p* < 0.001]. These findings indicate that the mice in which the VGlut2-dependent glutamatergic neurotransmission of the PV-expressing neurons was suppressed manifested behavioral dominance over their wild-type littermates.

#### Social interactions and reproduction

In the dyadic interactions, significant genotypic differences occurred in the total duration of body sniffing only between two females (not shown), being shorter in the *KO-* and the *HET-* than in the *CTR*-mice [*F*_(2, 9)_ = 6.66, *p* = 0.017; *CTR* vs. *HET p* = 0.042, *HET* vs. *KO p* = 0.89, *CTR* vs. *KO p* = 0.020]. With respect to the other types of sniffing, as well as to the parameter of sniffing in general (Figure 6D) [*F*_(2, 9)_ = 2.12, *p* = 0.18; *CTR* vs. *HET p* = 0.66, *HET* vs. *KO p* = 0.51, *CTR* vs. *KO p* = 0.16], trends in the same direction were observed. In accord with our data on vocalizations, this finding suggests that the *KO-* and the *HET-*mice might be less socially motivated than the *CTR-*mice.

In the dyadic interactions between a male and a female, the total duration of the male's attempts to mount the females was shorter in the *KO-* than in the *CTR-*mice (Figure 8B) [*F*_(2, 55)_ = 5.03, *p* = 0.010; *CTR* vs. *HET p* = 0.106, *HET* vs. *KO p* = 0.49, *CTR* vs. *KO p* = 0.007]. However, whether the findings really reflected differences in sexual motivation or merely differences in general locomotor activity could not be ascertained.

Our attempts to interbreed the *KO-*mice generally failed. This finding suggests that they were less fertile than the *HET*-mice, which produced most of the pups used in the present study.

#### (Spatial) memory

The IntelliCage test afforded no evidence of the existence of memory deficits in the *KO-* and the *HET-*mice. On the contrary, they tended to perform even better than the *CTR*-genotype, making fewer errors in some of the tasks (Figures S3G,H) [PPRA: *F*_(1, 56)_ = 11.6, *p* = 0.001; PPRP: *F*_(1, 55)_ = 12.9, *p* < 0.001].

In the Morris water maze, none of the genotypes learned to find their way to the hidden platform. However, according to most of the measures of training performance (e.g., escape latency) (Figure S2A) [acquisition phase: *F*_(2, 20)_ = 6.17, *p* = 0.008; *CTR* vs. *HET p* = 0.02, *HET* vs. *KO p* = 0.47, *CTR* vs. *KO p* = 0.005; reversal phase: *F*_(2, 20)_ = 12.9, *p* < 0.001; *CTR* vs. *HET p* < 0.001, *HET* vs. *KO p* = 0.65, *CTR* vs. *KO p* < 0.001], the *KO-* and the *HET-*mice performed less well than the *CTR-*mice. Once again, an unambiguous interpretation of these findings is not possible, since the locomotor deficits of the *KO-* and the *HET-*mice might have contributed to their poor performance in this test. Particularly the tendencies of the *KO*-mice to swim in circles (Figure S2C) and to float on the surface of water (Figure S2D) [*F*_(2, 20)_ = 2.35, *p* = 0.12; *CTR* vs. *HET p* = 0.59, *HET* vs. *KO p* = 0.11, *CTR* vs. *KO p* = 0.028] increased the likelihood of their requiring more time than the *CTR-*mice to locate the platform. We also observed that the *KO-* and the *HET*-mice hit the platform without subsequently attempting to climb onto it. Factors such as the degree of motivation or of exhaustion may have been of relevance in this context.

On the basis of the data gleaned from the IntelliCage test and the Morris water maze, we presume that the *KO-* and the *HET-*mice manifested no signs of deficits in memory.

#### Anxiety and stress

The parameters deemed to reflect anxiety or stress in the elevated zero maze revealed no consistent differences between *KO*-mice and their wild-type littermates (Figure S4) [entries into open sectors: *F*_(1, 23)_ = 9.68, *p* = 0.005 [*CTR*>*MUT*]; time in open sectors: *F*_(1, 23)_ = 0.01, *p* = 0.91; unprotected head dips: *F*_(1, 23)_ = 0.95, *p* = 0.34; stretched attend postures: *F*_(1, 23)_ = 6.47, *p* = 0.02 [*CTR*>*MUT*]; fecal boli deposited: *F*_(1, 23)_ = 10.60, *p* = 0.003 [*CTR*>*MUT*]]. In accordance with this, thigmotaxis in the Morris water maze did not differ between genotypes (not shown).

## Discussion

We generated a mouse line in which the expression of the *Slc17a6* gene, which encodes the vesicular glutamate transporter protein VGlut2, was selectively eliminated from PV-expressing neurons. These PV-Cre;VGlut2-Lox mutant mice allowed us to study the phenotypic consequences of eliminating VGlut2-dependent glutamatergic neurotransmission in this neuronal population. The efficacy of such a Cre-Lox approach, used in combination with *Slc17a6-Lox* mutant mice, has been confirmed previously (Tong et al., 2007; Wallén-Mackenzie et al., 2009; Birgner et al., 2010). Knocking out VGlut2 in PV- neurons with the Cre/Lox technique leads to a loss of ~17% of this neurotransmitter transporter in the brain, and this decrease in concentration could be documented pictorially in individual brain nuclei rich in PV/VGlut2 terminals (e.g., Su3 of the PAG). Since most PV-expressing neurons in the central nervous system use GABA as a neurotransmitter, their function remains intact. In contrast, the entirety of neurons that rely solely on VGlut2 to load the synaptic vesicles with glutamate, and that in addition express PV, are deficient in glutamatergic neurotransmission. However, not all these PV/VGlut2 neurons are functionally silenced; those that co-express GABA-markers (GAD and VGaT) may still release GABA from nerve terminals (Root et al., 2014). Out of the 14 brain regions that may co-express the mRNAs of both *Pvalb* and *Slc17a6* (see Table S1), the medial vestibular nucleus is the only region, in which the same neuron express markers of GABA-ergic neurotransmission (GAD or VGaT) (screening of the Allen Brain Atlas, Table S1). It is thus conceivable, that in PV-Cre;VGlut2-Lox mutant mice, medial vestibular neurons that express PV are deficient in glutamatergic-, but are still capable of GABA-ergic neurotransmission. This fact may influence the control of posture, thus contributing to the difficulties in locomotion observed in the PV-Cre;VGlut2-Lox mice (see section Locomotion).

In these “dual neurotransmitter” neurons, co-transmission of two “antagonistic” compounds involves spatial segregation of vesicles (Hattori et al., 1991; El Mestikawy et al., 2011; Vaaga et al., 2014), encoding of parallel signals that operate along different timescales (El Mestikawy et al., 2011) and differential release (Burnstock, 2007), depending on Ca^2+^ sensitivities (Vaaga et al., 2014). Co-released transmitters may also influence distinct target neurons (Nusbaum et al., 2001) and the packaging- (El Mestikawy et al., 2011) and release of one neurotransmitter may also be modulated by the other (Docherty et al., 1987). The deletion of VGluts in monoamine and cholinergic neurons leads to altered anxiety-related behaviors, sensitivity to psychostimulants and locomotion (Birgner et al., 2010; El Mestikawy et al., 2011).

The aims of the present study were to characterize the phenotype of the PV-Cre;VGlut2-Lox mutant mice using a battery of behavioral tests, and to investigate the brain regions which may underlie these phenotypic traits, of which the most conspicuous included deficits in locomotion and vocalization, decreased sensitivity to thermal pain and increased dominance.

### Locomotion

The observed deficits in locomotion may stem from disturbances in motor centers that rely on VGlut2 for synaptic transmission (e.g., the red, the subthalamic or the deep cerebellar nuclei) (Table S1), or from a predominance of GABA-ergic transmission, e.g., in the neurons of the medial vestibular nucleus, which normally mediates body posture. Alternatively, some locomotor deficits could arise indirectly, via a disturbance in motivational drive. The wobbly gait might be attributed to a failure in kinesthetic sensibility, which would be a consequence of the silencing of neurons in the gracile and the cuneate nuclei (Table S1) that depend on VGlut2 to process proprioceptive information arriving from the periphery. The VGlut2-positive ventral anterolateral complex of the thalamus (Table S1), which is involved in the coordination and planning of movement might also be involved. In the Morris water maze, *KO* mice tended to float on the surface of the water. Although this trait probably reflects a motor deficit, it could also be attributable to a lack of energy (exhaustion) or motivation to find the platform and escape from the water.

### Ultrasonic vocalization

On the basis of data gleaned from experiments with squirrel monkeys, two separate vocal pathways have been proposed. The limbic pathway, which relays in the hypothalamus (Velley, 1986; Wild et al., 2003) and PAG (Carrive and Morgan, 2012), is involved in non-verbal emotional vocal expression (Jürgens and Zwirner, 1996). It is also presumed to control the amplitude, pitch and intonation of vocalizations (Davis et al., 1996). The role of the hypothalamus in the limbic vocal pathway might be a modulation of the preparedness to vocalize (Dujardin and Jurgens, 2006). The PAG, on the other hand, has been postulated to participate in the induction of relaxed emotional states (Jürgens, 1991). Not only primates, but also mice produce emotional vocal expressions. Ultrasonic vocalizations of mice—both interaction-induced and female-induced—are today believed to not only bear a communicative signal facilitating the establishment of social contacts (Panksepp et al., 2007; Portfors, 2007; Hammerschmidt et al., 2009; Scattoni et al., 2011), but also to reflect a positive affective state (Panksepp and Lahvis, 2007; Panksepp et al., 2007; Wang et al., 2008; Malkesman et al., 2010).

In our study, we found PV-Cre;VGlut2-Lox mutant mice to vocalize less than control mice (mean call activity). Moreover, whereas female pairs of *CTR* and *HET* mice emitted USV of all 11 types that we distinguished, female pairs of KO mice produced only five types characterized by less complex patterns (short, flat, upward, downward, and chevron calls). Both the reduced call activity (Lahvis et al., 2011) and the reduced spectrum of vocalization patterns (Chabout et al., 2012) observed in PV-Cre;VGlut2-Lox mutant mice appear to indicate reduced positive affect and/or a reduced level of social motivation.

The neurons of the hypothalamic parvafox nucleus that are functionally connected to the limbic pathway of vocalizations and project to the PAG (Celio et al., 2013; Bilella et al., 2016) have presumably been impacted by the abolishment of the glutamatergic signaling, which could explain the observed deficits in vocalization. This hypothesis is supported by findings from our own previous studies, which revealed tickled rats to produce significantly fewer 50-kHz calls after excitotoxic lesioning of the parvafox nucleus than before (Roccaro-Waldmeyer et al., 2016). Alternatively, the observed deficits in vocalization might result from a deficit in the fine motoric control of the laryngeal musculature, caused by a disruption of VGlut2 dependent glutamatergic transmission in corresponding motor centers (Table S1).

### Pain

An adequate interpretation of findings appertaining to the perception of pain in the hot plate test could conceivably be hampered by deficits in locomotion. However, unlike in the Morris water maze, the *KO-* and the *HET-*mice responded to the stimulus in a manner indistinguishable from that in *CTR-*mice. Furthermore, the administration of drugs that impair motor performance neither prolongs the endpoint latencies in the hot plate test nor interferes with the effect of morphine on this parameter (Plummer et al., 1991). We therefore conclude that an increase in the threshold for the perception of pain, and not a deficit in locomotion, was responsible for the delayed responses of the *KO-* and the *HET-*mice to the heat stimulus.

The hot plate test is presumed to measure the central (supraspinal) rather than the spinal components of the pathways that contribute to nociception (Caggiula et al., 1995; Bourne et al., 2014). Only three of the structures implicated in nociception pathways were revealed in the survey of the Allen Brain Atlas to potentially co-express PV and VGlut2, namely, the PAG, the anterior pretectal and the ventral posterior nucleus of the thalamus, the last of which relays information borne by spinothalamic pain afferents to the somatosensory cortex. Although the ventral posterior nucleus is listed in Table S1 as a site in which both PV- and VGlut2-expressing neurons occur, the putatively double-labeled population is comparatively small. An involvement of the anterior pretectal thalamic nucleus is also unlikely, since the mRNA expression patterns of *Pvalb* and *Slc17a6* are not suggestive of substantial co-localization. Moreover, activation of the anterior pretectal thalamic nucleus results in anti-nociception (Rees and Roberts, 1993). Hence, any interference with its function would lower not raise the threshold for the perception of pain. This rational process of elimination leaves the PAG, the lateral and ventrolateral quadrants of which play an important role in processing and mediating the responses to somatic and visceral noxious stimuli (Mouton and Holstege, 1998). These lateral and ventrolateral quadrants are the terminus of inputs that stem from the lateral hypothalamic parvafox nucleus (see Figure 2), that co-express PV and VGlut2 (Celio et al., 2013; Bilella et al., 2016), and that are thus functionally silenced in PV-Cre;VGlut2-Lox mutant mice. It remains to be established whether the imbalance caused by the absence of the hypothalamic input contributes to the elevated threshold for the perception of thermal pain that was observed in the *KO-* and the *HET-*mice.

### Aggression and dominance

The tube test was first introduced as a model to measure dominance in 1961 (Lindzey et al., 1961), and it is still considered superior to other approaches that have since been elaborated to identify social hierarchies (Wang et al., 2014). The hierarchical ranks established by our mice remained very stable over the seven test days. In a previous study, identical ranking positions were achieved on two subsequent test days in 59% of cases (Wang et al., 2011). In our group of animals, the prevalence was 87% (65 out of 75 cases). The medial prefrontal cortex, which is a source of afferents to the lateral hypothalamus, has been implicated in behavioral traits related to the hierarchy of dominance (Zink et al., 2008; Chiao, 2010; Wang et al., 2011). Other regions that are implicated include all other areas of the prefrontal cortex, portions of the occipitotemporal and the parietal cortices, and subcortical structures, such as the amygdalar and the dorsal raphe nuclei, the hypothalamus and the PAG (Wang et al., 2014). On the basis of our survey of the Allen Brain Atlas, the anterior amygdalar area and small assemblages of neurons in many cortical zones putatively co-express PV and VGlut2, and may thus contribute to the phenotypical trait of dominance in the PV-Cre;VGlut2-Lox mutant mice. The hypothalamus and the PAG are implicated in defense and aggression (Hess, 1949) and as well listed in Table S1 as possible sites in which subpopulations of neurons co-express PV and VGlut2. The lateral PAG is the terminus of projections from the hypothalamic parvafox nucleus (Bilella et al., 2016), and it has long been thought to be the seat of defensive behavior in threatening situations (Bandler and Keay, 1996; Bandler et al., 2000). It is conceivable that the superior performance of the *KO-* and the *HET-*mice in the dominance-gauging tube test may be associated with functional competence of this lateral hypothalamic nucleus.

### Social interaction

Data gleaned from the dyadic interactions suggested that *KO-*mice might be less socially orientated than *CTR-*mice. However, in the three-chamber test, no genotypic differences in either sociability or social novelty were observed. Although the three genotypes performed as expected during the sociability phase (more time spent in the side chamber with the social stimulus), none of them did so during the phase of social novelty (more time spent in the side chamber with the newly introduced mouse). Deficits in locomotion could conceivably have influenced the findings in the *KO-*mice. But in the face of this possibility is the negating circumstance that the total distance traveled did not differ between the genotypes. Hence, the three-chamber test afforded no evidence of genotypic differences in social intercourse. Other sociability tests that depend less heavily on locomotor performance would help to eliminate the ambiguities.

*KO-*mice built nests of inferior quality, as compared to *CTR*-mice. Laboratory mice generally value greatly nest-building material, and they are usually highly motivated to undertake this task (Roper, 1973; Van De Weerd et al., 1998; Jirkof, 2014). A reduced performance in nest-building could be indicative of a depressed state of well-being (Arras et al., 2007; Deacon, 2012; Jirkof et al., 2013; Jirkof, 2014), which, together with a lack of motivation, might account for the poorer quality of the nests built by the *KO*-mice.

### Novelty and limitations

To our knowledge, this study represents the first attempt toward a brain-wide description of the population of neurons co-expressing PV and VGlut2. Prior studies in this context were purely anatomic and have merely focused on one specific brain region (most often thalamic nuclei). This study provides evidence for further brain regions, among them motor and sensory areas, to also contain neurons co-expressing PV and VGlut2, and gives an idea of the functional significance of the entirety of these neurons.

The limitations of this study consist in the unfeasibility of a brain-wide direct demonstration of PV and VGlut2 co-expression on a cellular level, as well as in the unpredictability of the changes arising secondary to the mutation *per se*. As a consequence of the functional silencing of the co-expressing neurons, downstream neurons are deprived of glutamatergic input activity and thus expected to be affected as well. The nature of the changes caused to downstream neurons depends on many factors. Conceivable are changes in spine number and/or spine shape, in the distribution of postsynaptic glutamate receptors, and even in downstream signaling machinery (Yuste and Bonhoeffer, 2004; Turrigiano, 2008; Sigler et al., 2017). Furthermore, in those neurons with dual neurotransmission (glutamate and GABA) (Seal and Edwards, 2006; Zander et al., 2010; El Mestikawy et al., 2011; Vaaga et al., 2014), the deficiency in one neurotransmitter (glutamate) could lead to dominance of the remaining one (GABA).

## Conclusion

The locomotor deficits of the PV-Cre;VGlut2-Lox mutant mice most likely stem from one or several of the motoric brain structures that co-express PV and VGlut2. The vocalization deficits might likewise reflect a disturbance of the fine motor control of the laryngeal musculature; an interference with the limbic vocal pathway at the level of the hypothalamic-PAG axis could also play a role. The deficits in thermal nociception and possibly also the increase in social dominance are probably explained, at least partially, by an impaired control of the PAG by afferents that are deprived of VGlut2 in this line of mutant mice.

## Author contributions

DR-W designed and conducted the experiments, analyzed and interpreted results, and wrote the paper. DM conducted experiments, analyzed and interpreted results. EV designed and conducted experiments. LP designed and conducted experiments, analyzed and interpreted results. DW designed the experiments, analyzed and interpreted results and wrote the paper. FG and MC designed the experiments and wrote the paper.

### Conflict of interest statement

The authors declare that the research was conducted in the absence of any commercial or financial relationships that could be construed as a potential conflict of interest.

## Abbreviations

ABA: Allen Brain Atlas
*CTR*: control(s) (*PV*^+/+^*;VGlut2*^*Lox*/*Lox*^)
*F*: female
*HET*: heterozygous (*PV*^*Cre*/+;^*VGlut2*^*Lox*/*Lox*^)
*KO*: knock-out (*PV*^*Cre*/*Cre*^*;VGlut2*^*Lox*/*Lox*^)
*M*: male
*MUT*: mutants (*HET*+*KO*)
PAG: periaqueductal gray
PV: parvalbumin protein
*Pvalb*: gene encoding the protein parvalbumin
*Slc17a6*, gene encoding the protein Solute Carrier Family 17: Member 6 (= VGlut2)
USV, ultrasonic vocalizations;VGlut2, vesicular glutamate transporter 2: encoded by the gene *Slc17a6*.
