# Supplementary material for: Eliminating the VGlut2-Dependent Glutamatergic Transmission of Parvalbumin-Expressing Neurons Leads to Deficits in Locomotion and Vocalization, Decreased Pain Sensitivity, and Increased Dominance
Source: Front Behav Neurosci. 2018 Jul 18;12:146. doi: 10.3389/fnbeh.2018.00146 (PMC6058961; doi:10.3389/fnbeh.2018.00146)
Supplement: Supplementary file 1 [file Table_1.PDF]

Supplementary Table 1: **Potential co-expression of the *Pvalb* and *Slc17a6* genes in 14 brain sites: results from the ABA-screening.** All brain areas found in the ABA screen to show expression for both *Pvalb* and *Slc17a6* mRNA are listed together with the overall intensity for both genes rated as one to three [(+)-(+++)]. In the 14 regions highlighted in gray, we considered a co-expression as likely, at least in a subset of neurons of the respective area. Image series "Pvalb - RP_071204_01_E06 – coronal" and "Slc17a6 - RP_050921_01_E03 – coronal" of the ABA served as basis for comparison. Image numbers correspond to the level at which potential co-expression in the respective brain area occurs first (rostral end). Expression intensities reflect overall intensity within the respective area across all sections it spans. ABA: Allen Brain Atlas, *Pvalb*: gene encoding the protein parvalbumin, *Slc17a6*: gene encoding the protein Solute Carrier Family 17, Member 6 (= VGlut2: vesicular glutamate transporter 2)

brain part	area	abbreviation in ABA	*Pvalb* image	*Pvalb* expression	*Slc17a6* expression	*Slc17a6* image
Cerebral Cortex (CTX)	Anterior olfactory nucleus, external part	AONe	8/57	++	+++	8/115
Cerebral Cortex (CTX)	Cortical layers 2-5 of various areas	-	9/57	+++	+(+)	14/115
Cerebral Cortex (CTX)	Piriform area, pyramidal layer	PIR2	9/57	+++	++	14/115
Cerebral Cortex (CTX)	Dorsal peduncular area, layer 5	DP5	11/57	+	++	18/115
Cerebral Nuclei (CNU)	Lateral septal nucleus, rostral (rostroventral) part	LSr	16/57	+++	++	26/115
Cerebral Nuclei (CNU)	Diagonal band nucleus	NDB	16/57	+++	+(+)	26/115
Cerebral Nuclei (CNU)	Claustrum	CLA	16/57	+	+++	26/115
Cerebral Nuclei (CNU)	Substantia innominata	SI	16/57	++	+	26/115
Cerebral Nuclei (CNU)	Medial septal nucleus	MS	18/57	+++	+	30/115
Cerebral Nuclei (CNU)	Anterior amygdalar area	AAA	21/57	++	+	39/115
Brain stem (BS)	Lateral preoptic area	LPO	21/57	+	++	39/115
Cerebral Nuclei (CNU)	Triangular nucleus of septum	TRS	21/57	+	+++	39/115
Brain stem (BS)	Anterodorsal nucleus of Thalamus	AD	23/57	+	+++	43/115
Brain stem (BS)	Anterior hypothalamic nucleus	AHN	23/57	+	++(+)	43/115
Brain stem (BS)	Lateral hypothalamic area	LHA	23/57	+	++	43/115
Brain stem (BS)	Anterior cingulate area, ventral part, layer 5	ACAv5	23/57	+	++	43/115
Cerebral Nuclei (CNU)	Globus pallidus, internal segment	Gpi	25/57	+++	+	48/115
Brain stem (BS)	Ventral posteromedial nucleus of Thalamus	VPM	25/57	+	++(+)	48/115
Brain stem (BS)	Ventral posterolateral nucleus of Thalamus	VPL	25/57	+	++(+)	48/115
Brain stem (BS)	Ventral anterior-lateral complex of Thalamus	VAL	25/57	+++	++(+)	48/115
Brain stem (BS)	Paracentral nucleus of Thalamus	PCN	25/57	+(+)	++	48/115
Brain stem (BS)	Parvafox nucleus in LHA	-	26/57	+++	+++	53/115
Brain stem (BS)	Subthalamic nucleus	STN	29/57	+++	+++	59/115
Brain stem (BS)	Lateral mammillary nucleus	LM	32/57	++	+++	64/115
Brain stem (BS)	Supramammillary nucleus, lateral part	SUMl	31/57	+++	+++	63/115
Brain stem (BS)	Anterior pretectal nucleus of Thalamus	APN	32/57	++	+++	58/115
Cerebral Cortex (CTX)	Subiculum, dorsal part, pyramidal layer	SUBd-sp	33/57	++	++	62/115
Brain stem (BS)	Medial mammillary nucleus	MM	32/57	++	+++	64/115
Midbrain (MB)	Nucleus of Darkschewitsch	ND	36/57	+++	(+)	68/115

brain part	area	abbreviation in ABA	*Pvalb* image	*Pvalb* expression	*Slc17a6* expression	*Slc17a6* image
Midbrain (MB)	Interpeduncular nucleus	IPN	34/57	++	++	68/115
Brain stem (BS)	Medial geniculate complex, ventral part	MGv	33/57	++	+++	66/115
Midbrain (MB)	Red nucleus	RN	36/57	+++	+++	68/115
Midbrain (MB)	Midbrain reticular nucleus	MRN	35/57	+++	++	65/115
Hindbrain (HB)	Pontine gray	PG	35/57	+++	+++	72/115
Midbrain (MB)	Superior colliculus	SC	37/57	++	+++	70/115
Midbrain (MB)	Periaqueductal gray (lateral parts)	PAG	37/57	+	+++	73/115
Midbrain (MB)	Nucleus of the lateral lemniscus, ventral part	NLLv	39/57	+++	++	76/115
Hindbrain (HB)	Tegmental reticular nucleus	TRN	39/57	+++	++	76/115
Midbrain (MB)	Midbrain trigeminal nucleus	MEV	44/57	++(+)	++	85/115
Hindbrain (HB)	Pontine reticular nucleus, caudal part	PRNc	44/57	+++	+++	85/115
Midbrain (MB)	Inferior colliculus	IC	44/57	++	+++	85/115
Medulla (MY)	Ventral cochlear nucleus	VCO	44/57	+++	+++	85/115
Hindbrain (HB)	Principal sensory nucleus of the trigeminal	PSV	44/57	+++	+++	85/115
Medulla (MY)	Abducens nucleus	VI	45/57	+++	+	88/115
Hindbrain (HB)	Pontine central gray	PCG	45/57	++	++	88/115
Medulla (MY)	Gigantocellular reticular nucleus	GRN	45/57	++	++	88/115
Medulla (MY)	Magnocellular reticular nucleus	MARN	45/57	++	++	88/115
Medulla (MY)	Dorsal cochlear nucleus	DCO	47/57	+++	+	92/115
Cerebellum (CB)	Dentate nucleus of cerebellum	DN	47/57	+++	+++	92/115
Medulla (MY)	Intermediate reticular nucleus	IRN	47/57	++	+	92/115
Medulla (MY)	Nucleus prepositus	PRP	47/57	+++	+	92/115
Medulla (MY)	Superior vestibular nucleus	SUV	47/57	++	++	92/115
Cerebellum (CB)	Fastigial nucleus	FN	48/57	+++	+++	96/115
Cerebellum (CB)	Interposed nucleus	IP	48/57	+++	+++	96/115
Medulla (MY)	Lateral vestibular nucleus	LAV	48/57	++	++	94/115
Medulla (MY)	Medial vestibular nucleus	MV	48/57	+++	+++	94/115
Medulla (MY)	Paragigantocellular reticular nucleus, dorsal part	PGRNd	50/57	+++	++	100/115
Medulla (MY)	Spinal vestibular nucleus	SPIV	50/57	+	+	100/115
Medulla (MY)	External cuneate nucleus	ECU	52/57	+++	++	104/115
Medulla (MY)	Lateral reticular nucleus, magnocellular part	LRNm	52/57	+++	+(+)	104/115
Medulla (MY)	Cuneate nucleus	CU	52/57	++	+(++)	104/115
Medulla (MY)	Spinal nucleus of the trigeminal, caudal part	SPVC	53/57	+++	+	108/115
Medulla (MY)	Spinal nucleus of the trigeminal, interpolar part	SPVI	53/57	++	++	108/115
Medulla (MY)	Gracile nucleus	GR	56/57	+++	+++	113/115
Medulla (MY)	Medullary reticular nucleus, ventral part	MDRNv	55/57	++	++	110/115
