# Supplementary material for: Eliminating the VGlut2-Dependent Glutamatergic Transmission of Parvalbumin-Expressing Neurons Leads to Deficits in Locomotion and Vocalization, Decreased Pain Sensitivity, and Increased Dominance
Source: Front Behav Neurosci. 2018 Jul 18;12:146. doi: 10.3389/fnbeh.2018.00146 (PMC6058961; doi:10.3389/fnbeh.2018.00146)
Supplement: Supplementary file 2 [file Data_Sheet_1.docx]

Supplementary Material

Eliminating the VGlut2-dependent Glutamatergic Transmission of Parvalbumin-expressing Neurons leads to Deficits in Locomotion and Vocalization, Decreased Pain Sensitivity and Increased Dominance

Diana M. Roccaro-Waldmeyer^1^, Franck Girard^1^, Daniele Milani^1^, Elisabetta Vannoni^2^, Laurent Prétôt^1^, David P. Wolfer^2,3,4^, Marco R. Celio^1*^

*** Correspondence:** Marco R. Celio: [marco.celio@unifr.ch](mailto:marco.celio@unifr.ch)

Supplementary Materials and Methods

ActiviScope: Circadian Rhythm

To investigate circadian rhythm, two different groups of mice were used for ActiviScope experiments. ActiviScope 1 involved 20 female mice (11 *CTR*, 5 *HET* and 4 *KO*). ActiviScope 2 involved a total of 30 mice, both males and females (12 *CTR* [6*M*, 6*F*], 12 *HET* [7*M*, 5*F*] and 6 *KO* [2*M*, 4*F*]). In both experiments, home cage activity of individually housed mice was recorded using a rack of standard type II mouse cages (267 mm long x 207 mm wide x 140 mm high) equipped with one passive IR sensor per mouse (ActiviScope, New Behavior Inc., Zurich, Switzerland, www.newbehavior.com). Data were collected using proprietary software. The sensors detected any locomotion and remained silent only when the mice were sleeping or grooming. In order to optimize movement detection, no tubes or home boxes were added to the cages. ActiviScope 1 was composed of a first phase with standard light/dark cycle during 6 days, followed by a reversal phase of 22 days before an ultimate phase of constant darkness for 21 days. ActiviScope 2 consisted of only two phases, a standard light/dark cycle phase for 12 days followed by constant darkness during 15 days.

Morris Water Maze: Spatial Learning and Memory

This experiment involved the same 30 mice that were also used in ActiviScope 2. The apparatus and protocol are described elsewhere (Mohajeri et al., 2004). The acquisition phase consisted of 18 trials per animal (trials 1-18, platform location constant and identical for each of the four sample subsets) and was followed by a reversal phase of 12 trials per animal (trials 19-30), in which the hidden platform was moved to the opposite quadrant. The automatic tracking system provided by EthoVision (Noldus Information Technology) was used to assess parameters such as escape latency, swim speed, circling and floating behavior as well as thigmotaxis.

IntelliCage: Long-Term Cognitive Abilities

This experiment involved the same 20 mice that were also used later in ActiviScope 1. The IntelliCage apparatus (NewBehavior AG) is placed in a polycarbonate type III cage (20.5 cm high, 58 × 40 cm top, 55 × 37.5 cm bottom, Techniplast, 2000P, Buguggiate, Italy) and accommodates up to 16 mice. Its aluminum top contains a freely accessible food rack. The floor is covered with bedding and provides 4 central red shelters (Techniplast). Four triangular conditioning chambers (15×15×21 cm) are fitted in the cage corners and provide room for one mouse at a time. Each chamber contains two drinking bottles, accessible via two round openings (13 mm diameter) with motorized doors. Three multicolor LEDs are mounted above each door and the chamber ceiling contains a motorized valve for delivery of air puffs. Mice that access a chamber are identified by a circular RFID antenna at its entrance (30 mm inner diameter) and the duration of their visit is determined by both the antenna reading and a temperature sensor that detects the presence of the animal inside the corner. During a visit, number and duration of individual nosepokes at each door are recorded using IR-beam sensors. Licking episodes at each bottle are monitored using lickometers (duration of the episode, number of licks, total contact time). IntelliCages have individual controllers and are connected to a central PC running the software that permits to design and run experiments, as well as to analyze the recorded data (IntelliCage Plus, NewBehavior AG). Two such cages were used, accommodating 10 mice each. After an initial exploration period of 24 hours (EXP), a total of 9 different protocols were run. They are summarized in the table hereafter.

| protocol name | duration | description |
| --- | --- | --- |
| Free Adaptation Protocol (FAP) | 7 days | All doors to corners open, mice can explore the cage, enter corners and drink from each corner and side. |
| Nosepoke Adaptation Protocol (NPAP) | 7 days | All doors are closed but open upon the first nosepoke per corner visit for 5 seconds, allowing mice to drink. |
| Drinking Session Adaptation Protocol (DSAP) | 7 days | Like NPAP, but access to water is restricted to 11-12 am and 4-5 pm. |
| Place Preference Acquisition (PPRA) | 6 days | Only in one constant target corner, the first nosepoke per corner visit will lead to door opening for 5 seconds. |
| Place Preference Reversal Protocol (PPRP) | 8 days | Like PPRA, but with a constant target corner different from the one in PPRA. |
| Serial Reversion Protocol (SRP) | 4 days | The target corner changes in every drinking session. |
| Chaining Protocol  (CP) | 8 days | With every nosepoke in a correct corner, the correct corner changes in a CW or CCW direction. |
| Chaining Reversal Protocol (CRP) | 10 days | Reversal of CP. |
| Place Avoidance Protocol (PAP) | 8 days | Nosepokes in one incorrect target corner are punished with an air-puff. |

**Supplementary Table 1.** Summary of the nine protocols run in the IntelliCage experiment.

Elevated Zero Maze: Anxiety-Related Behaviors

The same 31 mice used for the nest assessment were tested for anxiety-related behaviors on the elevated zero maze according to methods described before (Madani et al., 2003). In brief, time spent within the two protected, the two unprotected and the four intermediate sectors, the number of entries into protected and unprotected sectors, and the total distance traveled by each mouse were recorded using an automatic tracking system provided by EthoVision (Noldus Information Technology). In addition, protected and unprotected head dips as well as stretched attend postures (SAP) were registered using the keyboard event-recorder function of EthoVision and the number of fecal boli deposited was counted at the end of the 10 minutes.

Three-Chamber Test: Sociability and Social Novelty

A group of 28 mice (10 *CTR* [5*M*, 5*F*], 11 *HET* [5*M*, 6*F*] and 7 *KO* [2*M*, 5*F*]) was tested for social interactions in a three-chambered apparatus as described in the original publication (Moy et al., 2004). In brief, the test consisted of a habituation phase (all chambers empty), a sociability phase (wire cages in side chambers, empty vs. stranger 1) and a social novelty phase (wire cages in side chambers, stranger 1 vs. stranger 2). Four ovariectomized *female* mice were used as stimulus mice after 24 hours of habituation to the wire cages. The habituation phase was slightly modified from the original protocol: its duration was 10 instead of 5 minutes and the doorways to both empty side chambers were kept open, which allowed us to determine whether there was any side preference already in the absence of the extra cues (wire cages and stranger mice). Times spent in each of the three chambers as well as total distance traveled were recorded using EthoVision (Noldus Information Technology) and user-defined chamber areas.

| nuclei with PV/VGlut2 expressing neurons | | | GAD | VGaT |
| --- | --- | --- | --- | --- |
| ventral anterior lateral complex | |  | no | no |
| parvafox |  |  | no | no |
| subthalamic nucleus | |  | no | no |
| red nucleus |  |  | no | no |
| pontine reticular nucleus, posterior part | | | rare cells | single cells |
| ventral cochlear nucleus | |  | single cells | rare single cells |
| principal sensory nucleus trigeminal ggl | | | no | single cells |
| dentate nucleus cerebellum | |  | single cells | many faint cells |
| fastigial nucleus |  |  | rare cells | no |
| interposed nucleus |  |  | single cells | many faint cells |
| lateral vestibular nucleus | |  | no | no |
| medial vestibular nucleus | |  | many cells | many cells |
| cuneate nucleus |  |  | no | no |
| gracile nucleus |  |  | not valuable | single cells |

**Supplementary Table 1: Search for the co-expression of GAD and VGaT in the 14 brain regions in which VGlut2 is potentially co-expressed by parvalbumin neurons.** The medial vestibular nucleus is the only region out of these 14, in which parvalbumin neurons probably express GABA-markers in addition to VGlut2.

| parameter (experiment) | sex effect | *F*: mean ± SEM | *M*: mean ± SEM | (genotype x sex) interaction |
| --- | --- | --- | --- | --- |
| body weight [g] | F_(1,1194)_=496.68, p<0.001 | 17.84 ± 0.15 g (n=66) | 22.41 ± 0.14 g (n=58) | F_(2,1193)_=9.41, p<0.001 |
| quality of nests | F_(1,29)_=0.14, p=0.72 | 3.67 ± 0.26 (n=14) | 3.80 ± 0.25 (n=17) | F_(2,28)_=0.76, p=0.48 |
| number of line crossings (open field) | F_(1,27)_=11.63, p=0.002 | 14.40 ± 4.06 (n=13) | 32.84 ± 3.57 (n=16) | F_(2,26)_=0.17, p=0.85 |
| exploratory activity - rearing (open field) | F_(1,27)_=5.63, p=0.03 | 3.49 ± 1.60 (n=13) | 8.56 ± 1.41 (n=16) | F_(2,26)_=1.08, p=0.36 |
| grooming (open field) | F_(1,27)_=0.53, p=0.47 | 1.69 ± 0.20 (n=13) | 1.88 ± 0.17 (n=16) | F_(2,26)_=0.86, p=0.44 |
| number of fecal boli deposited (open field) | F_(1,27)_=4.71, p=0.04 | 1.93 ± 0.35 (n=13) | 2.96 ± 0.31 (n=16) | F_(2,26)_=0.45, p=0.64 |
| latency to endpoint (hot plate 1) [s] | F_(1,25)_=0.01, p=0.91 | 29.18 ± 2.08 (n=13) | 28.82 ± 2.29 (n=14) | F_(2,24)_=0.53, p=0.59 |
| latency to endpoint (hot plate 2) [s] | F_(1,22)_=0.50, p=0.49 | 22.11 ± 2.23 (n=11) | 19.94 ± 2.11 (n=13) | F_(2,21)_=1.29, p=0.30 |
| homecage activity counts (ActiviScope) | F_(1,46)_=0.22, p=0.64 | 15.62 ± 1.08 (n=34) | 14.63 ± 1.83 (n=14) | F_(2,45)_=1.58, p=0.22 |
| escape latency (Morris water maze, acquisition) [s] | F_(1,19)_=0.28, p=0.60 | 96.08 ± 5.42 (n=13) | 91.76 ± 6.13 (n=13) | F_(2,18)_=1.21, p=0.32 |
| escape latency (Morris water maze, reversal) [s] | F_(1,19)_=1.81, p=0.19 | 94.08 ± 5.84 (n=13) | 82.23 ± 6.60 (n=13) | F_(2,18)_=0.03, p=0.98 |
| swim speed (Morris water maze) [m/s] | F_(1,19)_=3.05, p=0.10 | 0.129 ± 0.005 (n=13) | 0.143 ± 0.006 (n=13) | F_(2,18)_=0.07, p=0.94 |
| circling behavior (Morris water maze) [⁰] | F_(1,19)_=2.12, p=0.16 | 1406.4 ± 120.1 (n=13) | 1142.7 ± 135.6 (n=13) | F_(2,18)_=2.72, p=0.09 |
| entries into open sectors (elevated zero maze) | F_(1,25)_=1.86, p=0.19 | 16.01 ± 2.29 (n=14) | 20.48 ± 2.36 (n=13) | F_(1,25)_=6.60, p=0.02 |
| number of stretched attend postures (elevated zero maze) | F_(1,25)_=0.82, p=0.37 | 1.307 ± 0.203 (n=14) | 1.570 ± 0.209 (n=13) | F_(1,25)_=0.15, p=0.70 |
| number of fecal boli deposited (elevated zero maze) | F_(1,25)_=0.41, p=0.53 | 3.458 ± 0.462 (n=14) | 3.881 ± 0.476 (n=13) | F_(1,25)_=1.26, p=0.27 |

**Supplementary Table 2**. **Summary of Statistically Significant Sex Differences and of Statistical Interaction (genotype x sex).** For the experiments analyzed using two-way ANOVA with genotype and sex as between-subject factors, the studied parameters are indicated in separate lines, together with the F values and significance levels for the two between-subject factors, and mean ± SEM per sex.

Supplementary Figure Legends

**Supplementary Figure 1.** **Circadian activity: reduced home-cage activity in *KO* and *HET* but no deficiency in maintaining circadian rhythm.** Activity counts for mice of the three genotypes during regular dark/light rhythms (upper parts) and in constant darkness (lower parts) for ActiviScope 1 **(A)** and ActiviScope 2 **(B)**. White backgrounds indicate that light was on, whereas gray backgrounds indicate dark phases. In both cohorts of mice, activity counts were much lower in both *KO* and *HET* than in *CTR* mice. Only in the first cohort (ActiviScope 1), activity of the *HET* group was impressively disorganized in constant darkness **(A)**. This effect was not reproduced, however, by the *HET* group of cohort 2 **(B)**. *(n* = 11 *CTR*, 5 *HET*, 4 *KO* for ActiviScope 1, *n* = 12 *CTR*, 12 *HET*, 6 *KO* for ActiviScope 2).

**Supplementary Figure 2.** **Morris water maze: *KO* and *HET* perform more poorly than *CTR*, showing reduced swim speed and increased circling behavior.** While none of the three genotypes showed significant spatial learning, *KO* and *HET* mice had even higher escape latencies than *CTR* mice during trials of both the acquisition (1-18) and the reversal phase (19-30) **(A)**. *KO* mice swam slower than both *HET* and *CTR* mice **(B)**. Circling behavior was much more pronounced in *KO* and *HET* than in *CTR* mice and during acquisition than during reversal **(C)**. Finally, *KO* tended to float longer on the surface than *CTR* mice **(D)**. In **(B-D)**, empty boxes and boxes filled in light and dark gray represent data of *CTR*, *HET* and *KO* mice, respectively. (*n* = 12 *CTR*, 12 *HET*, 6 *KO*). (# p < 0.05, ## p < 0.01, ### p < 0.001).

**Supplementary Figure 3.** **IntelliCage: *KO* removal due to insufficient drinking, *MUT*-mice superior in place preference reversal protocol, no learning deficits in *MUT* mice.** Data of *HET* and *KO* are analyzed and displayed together as data of the mutant group (*MUT*). *MUT* mice visited the corners less often than *CTR* mice, amongst others during free adaptation **(A)**. *MUT* mice made more nosepokes per corner visit **(B)**, stayed longer in the corners **(C)**, and licked less **(D)** than *CTR* mice. *MUT* mice showed stronger spontaneous left/right side preferences **(E)**, and during free adaptation also stronger spontaneous corner preferences than *CTR* **(F)**. Both genotypes learned the tasks as expected, and *MUT* mice tended to perform even better than *CTR* mice by making less errors in some tasks, for example during the place preference acquisition **(G)** or in the place preference reversal protocol **(H)**. (*n* = 11 *CTR*, 5 *HET*, 4 *KO*). DSAP: drinking session adaptation protocol, EXP: initial exploration period, FAP: free adaptation protocol, NPAP: nosepoke adaptation protocol, PPRA: place preference acquisition protocol, PPRP: place preference reversal protocol. (# p < 0.05, ## p < 0.01, ### p < 0.001).

**Supplementary Figure 4.** **Elevated zero maze: no significant genotype differences in anxiety-related behavior.** Data of *HET* and *KO* are analyzed and displayed together as data of the mutant group (*MUT*). *MUT* (especially the *males*) made less entries into open sectors than *CTR* **(A)**, however both spent comparable times in open sectors **(B)**. We found comparable numbers of unprotected head dips **(C)**, but less stretched attend postures **(D)** as well as less fecal boli **(E)** in *MUT* than in *CTR*. Both groups traveled comparable distances during the 10 minutes **(F)**. Boxes filled in light and dark gray represent data of *female* and *male* mice, respectively. (*n* = 12 *CTR*, 15 *MUT*). (## p < 0.01).

**Supplementary Figure 5.** **Three-chamber test: no significant genotype differences in sociability or social novelty.** **(A-C)** summarize the side preference per genotype and sex, whereas **(E-G)** show how much time mice of the three genotypes spent in the three chambers for each experimental phase. In **(A-C)**, boxes filled in light and dark gray represent data of *female* and *male* mice, respectively, whereas in **(E-G)**, data of *female* and *male* mice are pooled together. During the habituation phase, only the *KO females* had a significant preference for one of the two still empty chamber sides **(A)**. Overall, there was no genotype difference in side preference during habituation **(A,E)**. During the sociability phase, mice of both sexes spent more time in the side chamber with the social stimulus than in the chamber with the empty wire cage, independent of genotype **(B,F)**. During the social novelty phase however, none of the genotypes spent more time in the chamber with the newly inserted unfamiliar stimulus mouse than in the chamber with the already familiar one **(C,G)**. Total distance traveled did not differ between genotypes but was higher in *males* than in *females* **(D,H)**. (*n* = 10 *CTR*, 11 *HET*, 7 *KO*). (# p < 0.05).

Madani, R., Kozlov, S., Akhmedov, A., Cinelli, P., Kinter, J., Lipp, H.P., et al. (2003). Impaired explorative behavior and neophobia in genetically modified mice lacking or overexpressing the extracellular serine protease inhibitor neuroserpin. *Molecular and cellular neurosciences* 23(3)**,** 473-494.

Mohajeri, M.H., Madani, R., Saini, K., Lipp, H.P., Nitsch, R.M., and Wolfer, D.P. (2004). The impact of genetic background on neurodegeneration and behavior in seizured mice. *Genes, brain, and behavior* 3(4)**,** 228-239. doi: 10.1111/j.1601-1848.2004.00073.x.

Moy, S.S., Nadler, J.J., Perez, A., Barbaro, R.P., Johns, J.M., Magnuson, T.R., et al. (2004). Sociability and preference for social novelty in five inbred strains: an approach to assess autistic-like behavior in mice. *Genes, brain, and behavior* 3(5)**,** 287-302. doi: 10.1111/j.1601-1848.2004.00076.x.
