# Supplementary figures and images for: Eliminating the VGlut2-Dependent Glutamatergic Transmission of Parvalbumin-Expressing Neurons Leads to Deficits in Locomotion and Vocalization, Decreased Pain Sensitivity, and Increased Dominance
Source: Front Behav Neurosci. 2018 Jul 18;12:146. doi: 10.3389/fnbeh.2018.00146 (PMC6058961; doi:10.3389/fnbeh.2018.00146)

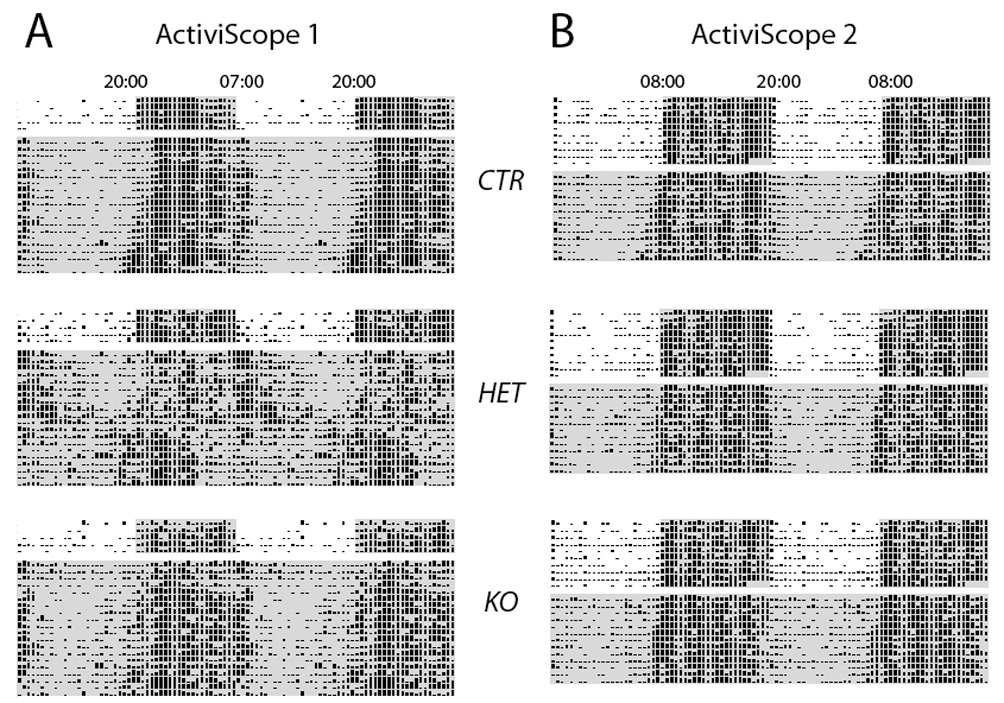

Supplement: Supplementary file 3 [file Image_1.JPEG]

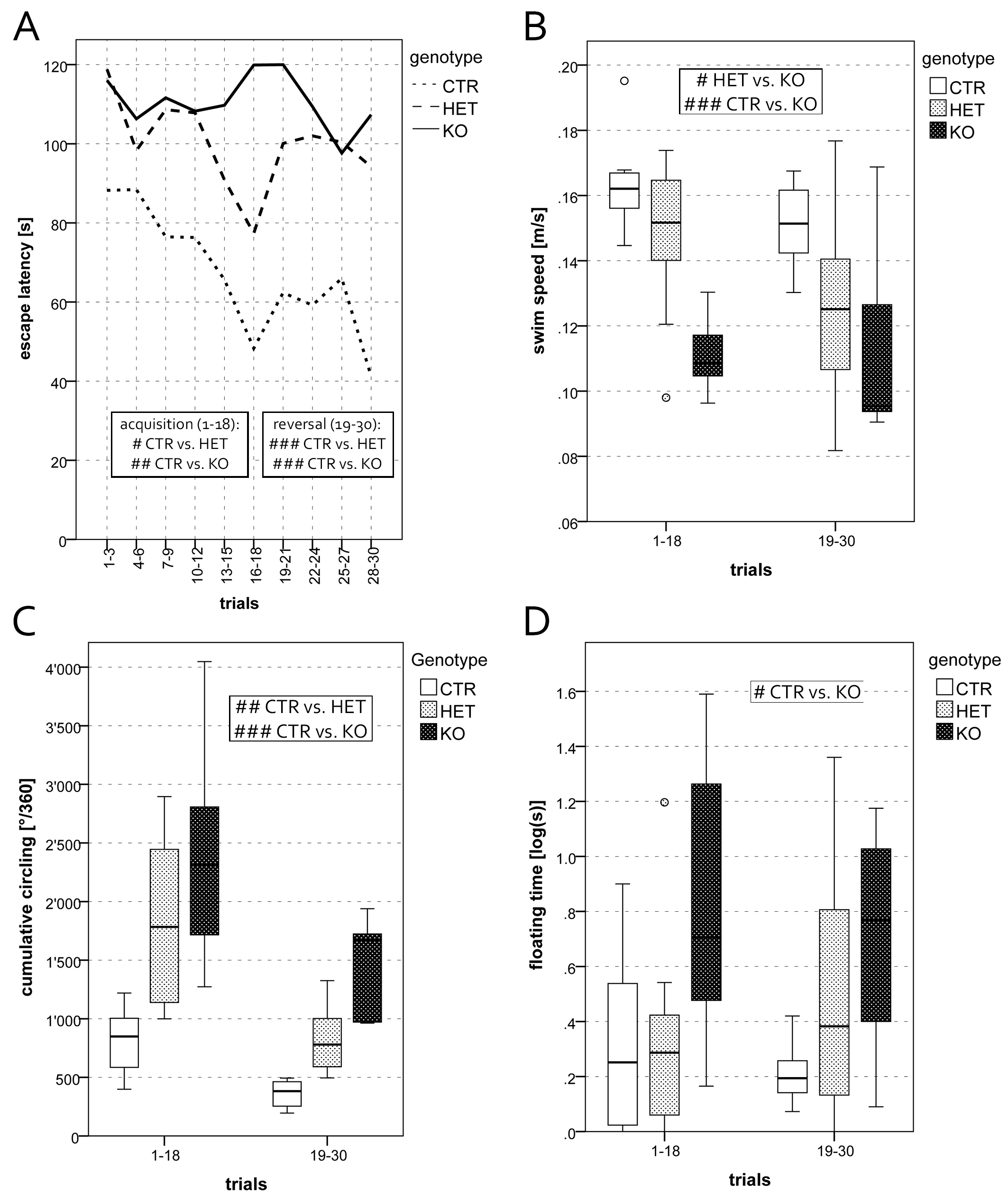

Supplement: Supplementary file 4 [file Image_2.JPEG]

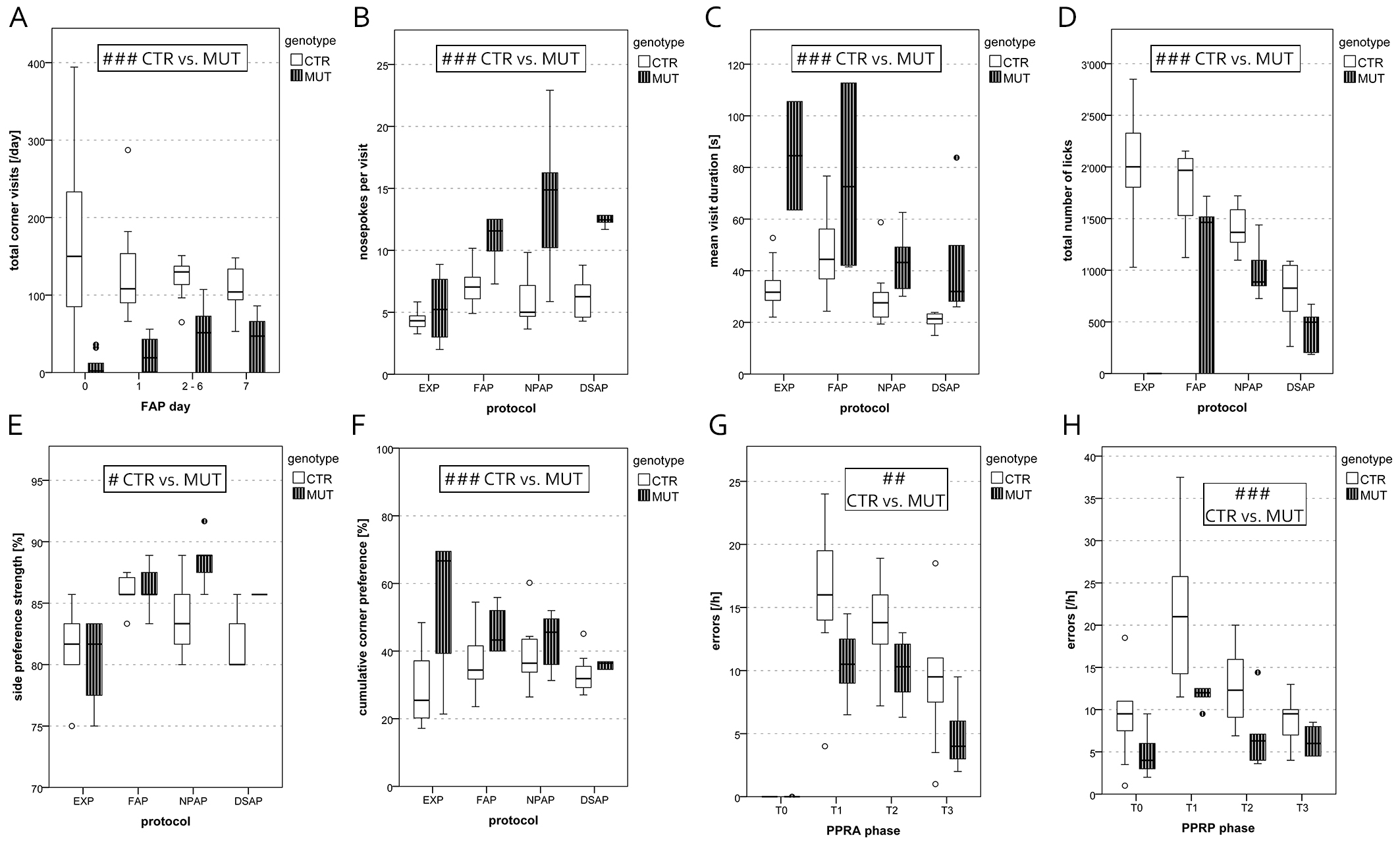

Supplement: Supplementary file 5 [file Image_3.JPEG]

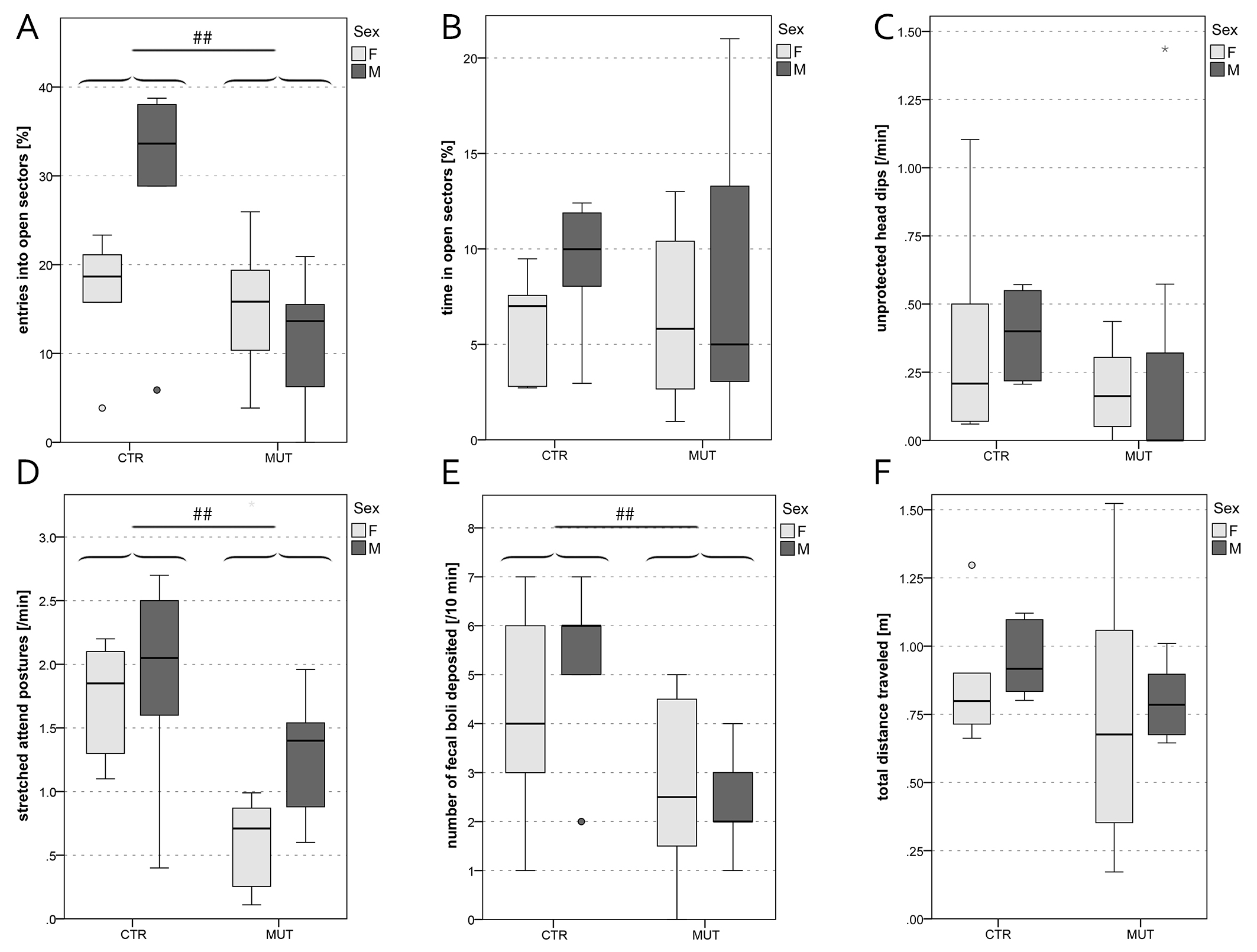

Supplement: Supplementary file 6 [file Image_4.JPEG]

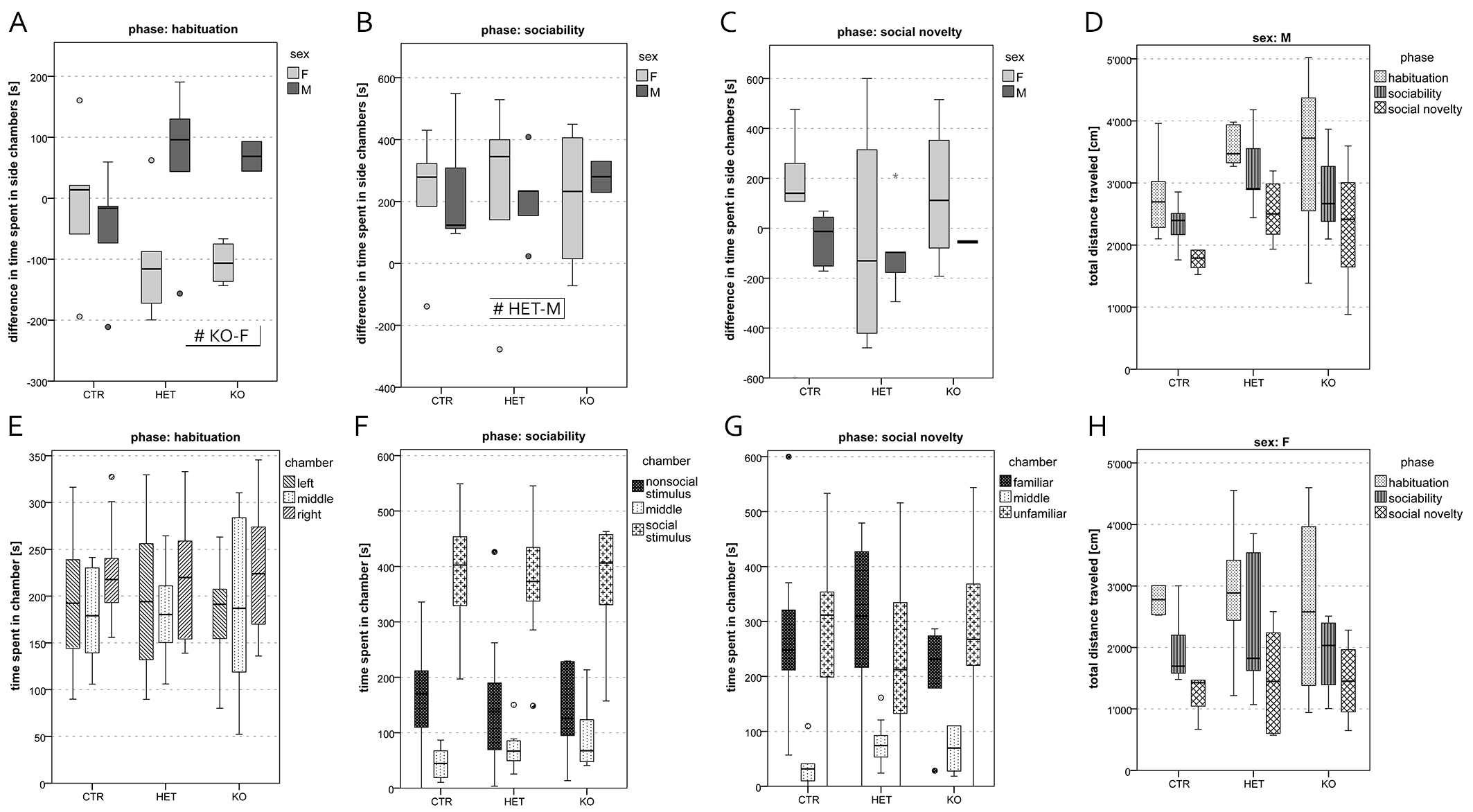

Supplement: Supplementary file 7 [file Image_5.JPEG]
